# The prognostic value of gastroesophageal reflux disorder in interstitial lung disease related hospitalizations

**DOI:** 10.1186/s12931-023-02407-4

**Published:** 2023-03-30

**Authors:** Shehabaldin Alqalyoobi, Bertis Brit Little, Justin M. Oldham, Ogugua Ndili Obi

**Affiliations:** 1 Department of Pulmonary and Critical Care Medicine, East Carolina University, Greenville, NC USA; 2 Department of Bioinformatics and Biostatistics, School of Public Health and Information Sciences, University of Louisville, Louisville, KY USA; 3 Department of Pulmonary and Critical Care Medicine, University of Michigan Medicine, Ann Arbor, MI USA

**Keywords:** Gastroesophageal reflux disease, Interstitial lung disease, In-hospital mortality, Respiratory failure

## Abstract

**Background:**

Gastroesophageal reflux disease (GERD) is a common comorbidity in patients with interstitial lung disease (ILD). We built and validated a model using the national inpatient sample (NIS) database to assess the contributory role of GERD in ILD-related hospitalizations mortality.

**Methods:**

In this retrospective analysis, we extracted ILD-related hospitalizations data between 2007 and 2019 from the NIS database. Univariable logistic regression was used for predictor selection. Data were split into the training and validation cohorts (0.6 and 0.4, respectively). We used decision tree analysis (classification and regression tree, CART) to create a predictive model to explore the role of GERD in ILD-related hospitalizations mortality. Different metrics were used to evaluate our model. A bootstrap-based technique was implemented to balance our training data outcome to improve our model metrics in the validation cohort. We conducted a variance-based sensitivity analysis to evaluate GERD's importance in our model.

**Findings:**

The model had a sensitivity of 73.43%, specificity of 66.15%, precision of 0.27, negative predictive value (NPV) of 93.62%, accuracy of 67.2%, Matthews Correlation Coefficient (MCC) of 0.3, F1 score of 0.4, and area under the curve (AUC) for the receiver operating characteristic (ROC) curve of 0.76. GERD did not predict survival in our cohort. GERD contribution to the model was ranked the eleventh among twenty-nine variables included in this analysis (importance of 0.003, normalized importance of 5%). GERD was the best predictor in ILD-related hospitalizations who didn’t receive mechanical ventilation.

**Interpretations:**

GERD is associated with mild ILD-related hospitalization. Our model-performance measures suggest overall an acceptable discrimination. Our model showed that GERD does not have a prognostic value in ILD-related hospitalization, indicating that GERD per se might not have any impact on mortality in hospitalized ILD patients.

**Supplementary Information:**

The online version contains supplementary material available at 10.1186/s12931-023-02407-4.

## Introduction

Interstitial lung diseases (ILD) comprise a diverse group of parenchymal lung diseases characterized by varying degrees of lung inflammation and fibrosis [1]. The etiology of Idiopathic pulmonary fibrosis (IPF) [2] sarcoidosis is unknown, while connective tissue diseases–ILD (CTD-ILD) and hypersensitivity pneumonitis (HP) might have an identified trigger [1, 3]. Relationship between gastroesophageal reflux disorder (GERD) and idiopathic pulmonary fibrosis (IPF) has been described [4]. It is hypothesized that GERD is associated with chronic micro-aspiration that induces repetitive lung injury leading to pneumonitis, increased acid exposure, epithelial permeability, fibrotic hyperplasia, and ultimately pulmonary fibrosis [5, 6]. Some retrospective studies showed that GERD is associated with higher survival in IPF patients [7], who had GERD medications, and those who received a Nissen fundoplication procedure for GERD [8]. On the other hand, post hoc analysis from different randomized controlled trials (RCT) showed that antisecretory medications (proton pump inhibitors and histamine-2 receptor blockers) were not associated with a more favorable course of the disease [9, 10]. The recent guidelines do not support treating IPF patients with antacid medication to improve IPF-related outcomes [11]. For sarcoidosis, a retrospective study showed that GERD is associated with decreased mortality [12]. As in IPF, It is also postulated that GERD can induce lung injury with similar mechanisms in CTD-ILD [13] and HP [14]. However, these studies included small numbers of patients and primarily targeted patients in the outpatient world.

Our study aimed to assess the contributory role of GERD in mortality in hospitalized patients with different groups of ILD (IPF, CTD-ILD, HP, and sarcoidosis) using a supervised machine learning (SML) approach.

## Methods

### Database

We extracted data from 2007 to 2019 from the national inpatient sample (NIS) database. This is the largest publicly available all-payer inpatient care database in the United States. The Agency for Healthcare Research and Quality (AHRQ) developed this database and is designed to estimate national inpatient outcomes [15]. The quality of the data is assessed annually [16]. Institutional Review Board approval for this study was not required as all patient data have been de-identified. Weighted, it estimates more than 35 million admissions every year. It utilizes the ninth revision of the International Classification of Diseases (ICD-9) codes through September 2015 and the tenth revision of the International Classification of Diseases (ICD-10) codes after that date.

### Case definition

First, we included patients with IPF codes (ICD-9 code 516.31 and ICD-10 code J84.112) and HP codes (ICD-9 codes 495.XX and 50.XX; and ICD-10 codes: J6.XX and J7.XX, except codes for radiation induced lung disease J70.0, J70.1, 508.0 and 508.1; and mycobacterial related infection J65 and 505). For sarcoidosis, we first isolated codes (ICD-9 code 135 and ICD-10 codes: D86.XX). Then, to capture all pulmonary sarcoidosis cases, we included patients who carried sarcoidosis codes along with codes of interstitial lung diseases defined by ICD-9 codes 517.8 (Lung involvement in other diseases classified elsewhere), and ICD-10 codes D86.0 (sarcoidosis of the lung), D86.1 (Sarcoidosis of lymph nodes) or D86.2 (Sarcoidosis of the lung with sarcoidosis of lymph nodes). For CTD-ILD, we only included patients with rheumatoid arthritis-ILD (RA-ILD), dermatomyositis/polymyositis-ILD (DMPM-ILD), and scleroderma-ILD (SSc-ILD). More details on CTD-ILD selection are explained in Additional file 1: Table S1. Cases with more than one diagnosis from the above categories (IPF, HP, CTD-ILD, or Sarcoidosis) are included as “Unspecified” ILD (Unspecified-ILD). For GERD, we included patients with ICD-9 codes 530.11 and 530.81, and ICD-10 codes K21.0 and K21.9. We excluded patients younger than 20 years old and those with lung transplants. For each case, we collected baseline demographic data (age, race, and sex), smoking status, hospital length of stay (HLoS), ILD subtypes (IPF, CTD-ILD, HP, pulmonary sarcoidosis, unspecified-ILD), and relevant comorbidities including chronic obstructive lung disease (COPD), asthma, respiratory failure (acute, chronic, acute on chronic or non-specified), obstructive sleep apnea (OSA), Barrett’s esophagus, hiatal hernia (HH), low body mass index (LBMI, BMI < 20), frailty, pneumonia, pulmonary hypertension (pHTN), obesity, acute pulmonary embolism (PE), and dependency on long term oxygen therapy (LTOT). We also gathered inpatient data procedures (including bronchoscopy and mechanical ventilation (MV) (invasive, non-invasive, both)), clinical classifications software codes [17], Elixhauser comorbidities [18, 19], and hospital region, location, size, and setting (Additional file 1: Tables S2, S3).

### Outcomes

The primary outcome assessed was inpatient all-cause mortality, defined as death during hospitalization.

### Statistical analysis

#### Variables selection

Categorical variables are reported as counts and percentages and were compared using the Chi-square test. Continuous variables are reported as means with standard deviation and compared using Student’s t-test. First, we performed a weighted univariable logistic regression for the variables: age, gender, race, smoking history, ILD subtype, low BMI, obesity, GERD, OSA, frailty, respiratory failure, use of MV, bronchoscopy, urban vs. rural hospital location, academic hospital status, COPD, asthma, acute PE, pHTN, HH, Barrett’s esophagus, pneumonia, and dependence on LTOT. Variables whose p-value was < 0.05 were selected to be included in the classification and regression tree (CART) analysis. We conducted a weighted decision tree analysis (using CART algorithm) for our primary outcome. The steps of our analysis approach are shown in Additional file 1: Figure S1.

#### CART analysis

CART is a nonparametric method for multivariable data that can build a model which will classify patients into different categories [20]. We used mortality as our target field (dependent outcome). The split at each node will produce a child node that is purer than its parent node using the Gini impurity measure (purity criterion). We set the minimum decrease in impurity required to split the node at 0.0001. In this analysis, CART will help us understand the relationship between GERD and mortality in ILD-related hospitalizations in 2 major ways: (1) Profiling: it will show which subgroup would GERD diagnosis be the next “best” predictor, and (2) Prediction: it will show if GERD diagnosis would predict mortality. In addition, we evaluated the predictor importance in the model using the variance-based sensitivity analysis method [21, 22] which computes the reduction in the variance of the target outcome attributed to each predictor via sensitivity analysis. It will assign a score (using first-order sensitivity measure) to each predictor based on its contribution to the model. It will compare the predictors to those with the highest importance score by assigning a normalized importance score.

To test our model, we split our data randomly into training and validation sets using SPSS (60% and 40%, respectively). Metrics used to evaluate our model are sensitivity, specificity, precision, negative predictive value (NPV), Matthews Correlation Coefficient (MCC), F1 score, accuracy rate, risk estimation, and ROC curve. We used a bootstrap-based technique in the training set to handle data with the imbalanced outcome to improve our model performance using the ROSE package [23].

#### Additional analysis

Gain chart, index, model risk estimates and confusion matrices for training and validation cohorts were developed. Subgroup CART analysis was performed to assess if GERD would contribute to patient mortality differently among ILD types. Besides, we split the data based on time (training set: between 2011–2013, and validation set: between 2016–2019).

Our approach to missing values is explained in the supplementary materials (Additional file 1: Table S4). Statistical significance was defined as p < 0.05 unless stated otherwise. Statistical analyses were performed using SPSS (IBM SPSS Statistics for Mac, Version 28.0; Armonk, New York: IBM Corp Released 2019), and R Core Team (2022) [24].

## Results

### Baseline characteristics

**Fig. 1:**
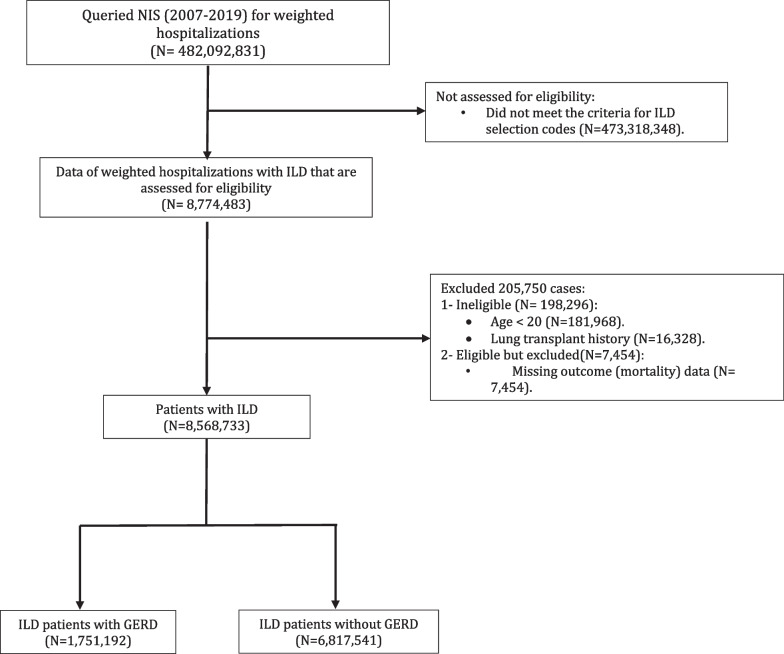
STROBE diagram (ILD: interstitial lung diseases; ICD: international classification of diseases; NIS: national inpatient sample; GERD: gastroesophageal reflux disease)

We identified 8,568,733 weighted hospitalizations with ILD between 2007 and 2019 that met the inclusion criteria (Fig. 1). 1,751,192 (20.4%) had GERD (Fig. 1). ILD patients with GERD were marginally older (71 ± 15 vs. 70 ± 17 years) and more likely to be smokers (30% vs. 25%). HP was the most common ILD subtype in the entire cohort (90.6%). Patients with IPF (4% vs. 2%), CTD-ILD (6% vs. 3%), pulmonary sarcoidosis (4% vs. 3%), and unspecified-ILD (1% vs 0.003%) were more likely to have GERD while patients with HP were less likely to have GERD (86% vs. 92%) (Table 1). ILD patients with GERD were more likely to have asthma, COPD, pHTN, OSA, obesity, Barrett’s esophagus, HH, and frailty (Table 1). ILD patients without GERD were more likely to be male, have respiratory failure, pneumonia, acute PE, lower BMI, and receive more bronchoscopy and invasive MV than ILD patients with GERD. While majority of the entire cohort was White, ILD patients who self-identified as non-White (Blacks, Hispanics, Asian or Pacific Islander, Native American) were less likely to have GERD while Whites were more likely to have GERD (72% vs. 79%).

**Table 1:** Hospitalizations characteristics for ILD patients with GERD vs. without GERD

	ILD patients with GERD (n = 1,751,192)	ILD patients without GERD (n = 6,817,541)	p-value
Age, mean ± SD	71.20 (15.26)	69.53 (17.19)	< 0.001
Age group (20–39), n (%)	67,245 (4)	477,374 (7)	< 0.001
Age group (40–59), n (%)	314,002 (18)	1,313,106 (19)	< 0.001
Age group (60–79), n (%)	743,468 (42)	2,620,494 (38)	< 0.001
Age group (80 or more), n (%)	625,867 (36)	2,405,310 (35)	< 0.001
Female, n (%)	841,440 (48)	2,826,688 (41)	< 0.001
*Race*			* < 0.001*
White, n (%)	1,298,312 (79)	4,558,471 (72)	
Black, n (%)	180,894 (11)	854,095 (14)	
Hispanic, n (%)	92,266 (6)	486,648 (8)	
Asian or Pacific Islander, n (%)	30,194 (2)	188,144 (3)	
Native American, n (%)	8218 (1)	37,738 (1)	
Other, n (%)	32,789 (2)	172,435 (3)	
*Smoking Status*			
Ever smoker, n (%)	529,037 (30)	1,698,764 (25)	< 0.001
Interstitial lung disease subtype:			< 0.001
Idiopathic pulmonary fibrosis, n (%)	63,756 (4)	126,550 (2)	
Connective tissue disease-ILD, n (%)	102,620 (6)	211,558 (3)	
Hypersensitivity pneumonitis, n (%)	1,506,754 (86)	6,258,846 (92)	
Pulmonary sarcoidosis, n (%)	66,706 (4)	198,014 (3)	
Unspecified-ILD, n (%)	11,354 (1)	22,574 (0)	
*Co-morbidities*			
Respiratory failure, n (%)	749,755 (43)	3,294,179 (48)	< 0.001
Dependence on long-term Oxygen, n (%)	161,134 (9)	353,694 (5)	< 0.001
Chronic obstructive lung disease, n (%)	211,515 (12)	655,650 (10)	< 0.001
Asthma, n (%)	166,251 (9)	364,574 (5)	< 0.001
Pneumonia, n (%)	265,203 (15)	1,105,779 (16)	< 0.001
Acute pulmonary embolism, n (%)	27,664 (2)	131,831 (2)	< 0.001
Pulmonary hypertension, n (%)	117,274 (7)	351,289 (5)	< 0.001
Obstructive sleep apnea, n (%)	171,273 (10)	377,630 (6)	< 0.001
Obesity, n (%)	202,655 (12)	612,569 (9)	< 0.001
Barrett's esophagus, n (%)	16,141 (1)	16,454 (0)	< 0.001
Hiatal hernia, n (%)	149,978 (9)	164,970 (2)	< 0.001
Low body mass index, n (%)	78,590 (4)	316,053 (5)	< 0.001
Frailty, n (%)	5519 (0.3)	16,374 (0.2)	< 0.001
*Elixhauser sum of conditions*			
Mean ± SD	2.31 (2.54)	2.46 (2.43)	< 0.001
Median (IQR)	2(4)	2(4)	
*Hospitalization Characteristics*			
Length of hospital stay, mean ± SD	8.21 (8.83)	10.40 (12.88)	< 0.001
*In-patient pulmonary Procedures*			
Bronchoscopy, n (%)	99,748 (6)	463,546 (7)	< 0.001
Invasive mechanical ventilation, n (%)	265,899 (15)	1,789,034 (26)	< 0.001
Non-invasive mechanical ventilation, n (%)	95,902 (5)	328,873 (5)	< 0.001
Mechanical ventilation (invasive and non-invasive), n (%)	26,682 (1.5)	143,626 (2.1)	< 0.001
*Bed size of hospital*			* < 0.001*
Small, n (%)	307,263 (18)	1,124,413 (17)	
Medium, n (%)	483,774 (28)	1,847,496 (27)	
Large, n (%)	955,684 (55)	3,824,916 (56)	
*Region of hospital*			* < 0.001*
Northeast, n (%)	326,379 (19)	1,373,817 (20)	
Midwest, n (%)	427,360 (24)	1,419,100 (21)	
South, n (%)	681,228 (39)	2,553,635 (37)	
West, n (%)	316,224 (18)	1,470,988 (22)	
Academic hospitals, n (%)	974,157 (56)	3,730,909 (55)	< 0.001
Urban hospitals, n (%)	1,540,479 (88)	6,098,589 (90)	< 0.001

### Univariable logistic regression

A univariable logistic regression of the variables: age, gender, race, smoking history, ILD subtype, low BMI, obesity, GERD, OSA, frailty, RF, use of MV, bronchoscopy, urban vs. rural hospital location, academic hospital status, COPD, asthma, acute PE, pHTN, HH, Barrett’s esophagus, pneumonia, and dependence on LTOT showed a p-value of < 0.05 (Additional file 1: Table S5), hence, all variables were selected for CART analysis.

### Training and validation datasets and handling imbalanced outcome data

After randomly assigning 60% of our data to a training set and 40% to a validation set, we performed our initial CART analysis. Our initial model’s metrics showed an accuracy of 85.5% and specificity of 100%. However, it showed poor performance on sensitivity (0%), precision, MCC, and F1 score (0) (Table 2). In this case, and because of our data’s imbalanced outcome (14.5% mortality vs. 85.5% survival), we proceeded with training data resampling to improve our classifier performance. More specifically, we oversampled the minority outcome group using the bootstrap-based technique. As a result, our new training data set had a balanced outcome (50% mortality and 50% survival). Baseline characteristics of the validation cohort were similar to the original data before splitting and resampling (Additional file 1: Tables S6, S7).

**Table 2:** Model metrics from the validation cohort before and after resampling of the training data

	Original data model	Resampled data model
Sensitivity (%)	0%	73.43%
Specificity (%)	100%	66.15%
Precision	N/A	0.2688
Negative Predictive Value (%)	85.50%	93.62%
Accuracy (%)	85.50%	67.20%
F1 Score	0	0.4
MCC	N/A	0.3
AUC(ROC)	0.75	0.76

F1 score = (2*precision*recall)/(precision + recall). MCC = TP*TN—FP*FN/sqrt((TP + FP)*(TP + FN)*(TN + FP)*(TN + FN))

### CART analysis

**Fig. 2:**
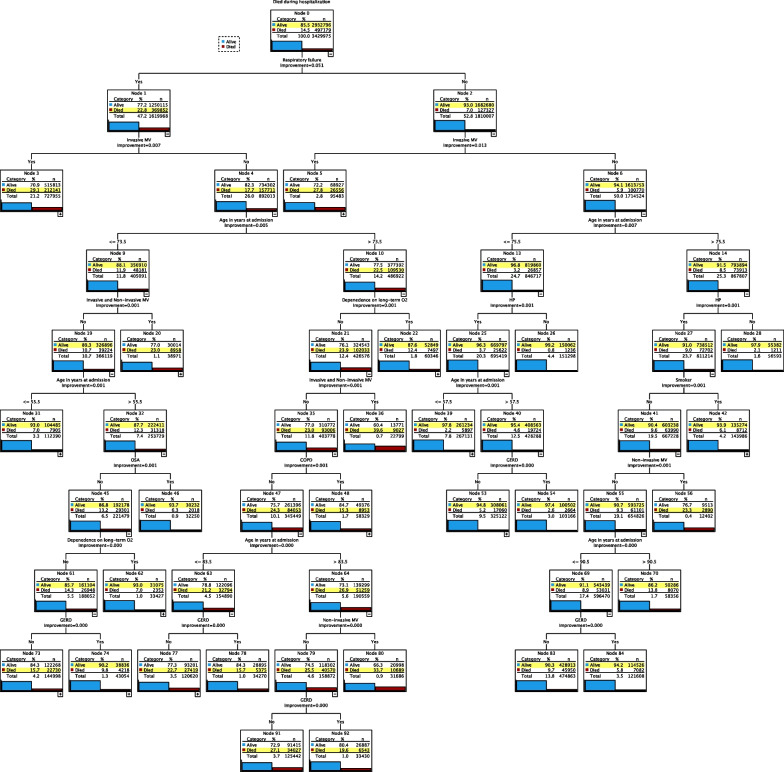
The decision tree for the validation cohort. The model performed well in predicting survival regardless of GERD (green) diagnosis [nodes 53&54 and 83&84] and poorly in predicting mortality regardless of GERD (red) diagnosis [nodes 91&92 and 77&78]. In one subgroup, our model performed well in predicting survival (green) [node 74] and poorly in predicting mortality (red) [node 73]. The bar chart in each node indicates the percentage of mortality. The predicted group is highlighted in yellow

**Fig. 3:**
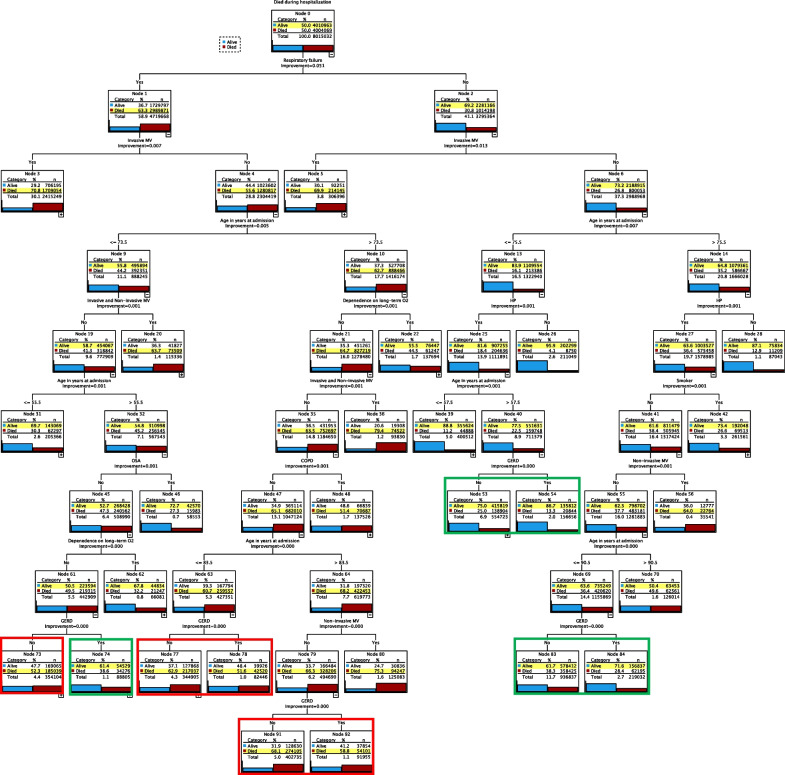
The decision tree for the training cohort (after resampling). The model performed well in predicting survival regardless of GERD (green) diagnosis [nodes 53&54 and 83&84] and mortality regardless of GERD (red) diagnosis [nodes 91&92 and 77&78]. In one subgroup, our model performed well in predicting survival (green) [node 74] and mortality (red) [node 73]. The bar chart in each node indicates the percentage of mortality. The predicted group is highlighted in yellow

Figures 2 and 3 depict the decision trees for the validation and the training samples respectively. They provide a graphical presentation for all predictors associated with mortality in descending order of importance. For the validation cohort, starting from the root node, in the overall sample of 3,429,975 patients, 85.5% of the patients survived the hospitalizations while the remaining 14.5% did not. GERD is selected as predictor in 4 parent nodes (53,73,74,77) and 6 terminal nodes (54,78,83,84,91,92). The decision starts with the root node that shows the distribution of the outcome field (i.e., mortality). The data is then split by the predictor with the strongest relationship, respiratory failure in this case. Patients with respiratory failure are split into patients who received invasive MV. In those who did not receive it, patients with an age of ≤ 73.5 years old are the next best predictor in this group. In this category, patients who did not receive both MV (invasive and non-invasive) and whose age is > 55.5 years are the best predictor. As we continue down this group path, we find that absence of OSA diagnosis and absence of long-term O2 therapy use are the next best predictor in this subgroup. Finally, GERD is the next best predictor in this cohort. Essentially, GERD (nodes 73 & 74) was found to be the best mortality predictor in hospitalized ILD patients aged 55.5 to 73.5 with respiratory failure, who did not receive invasive or both modes of MV, do not have OSA and were not on LTOT. In this subgroup, if patients did not have GERD (Node 73), we predicted that they would die 15.7% of the time. This prediction was applied to 144,998 patients and was accurate 22,730 times. If patients have GERD (Node 74), we predicted that they would survive 90.2% of the time. This rule was applied to 43,054 patients and was accurate 38,836 times. This indicates that diagnosis of GERD didn’t predict mortality or survival in ILD patients in this subgroup.

In the same decision tree, we can see that GERD (nodes 77& 78) is the best predictor in ILD patients who had respiratory failure, their age is (73.5–83.5), did not receive invasive or both modes of MV, did not have COPD and were not on LTOT. In this subgroup, GERD didn’t predict mortality. If patients did not have GERD (Node 77), we predicted they would die 22.7% of the time. This rule was applied to 120,620 patients and was accurate 27,419 times. If patients have GERD (Node 78), we predicted they would die 15.7% of the time. This prediction was applied to 34,270 patients and was accurate 5375 times.

In another branch, GERD (nodes 91&92) is the best predictor in ILD patients who had respiratory failure, their age is (> 73.5), did not receive invasive, non-invasive, or both modes of MV, did not have COPD, and were not on LTOT. In this subgroup, if patients did not have GERD (Node 91), we predicted that they would die 27.1% of the time. This rule was applied to 125,442 patients and was accurate 34,027 times. If patients have GERD (Node 92), we predicted that they would die 19.6% of the time. This rule was applied to 33,430 patients and was accurate 6543 times. This suggest that GERD didn’t predict mortality in this subgroup.

Another branch showed that GERD (nodes 53&54) didn’t predict survival. In this subgroup, GERD is the next best predictor in patients who did not have respiratory failure, did not receive invasive MV, had HP, and their age (57.5–75.5). In this subgroup, if patients did not have GERD (Node 53), we predicted that they would survive 94.8% of the time. This rule was applied to 325,122 patients and was accurate 308,061 times. If patients have GERD (Node 54), we predicted that they would survive 97.4% of the time. This rule was applied to 103,166 patients, and we were accurate 100,502 times.

In the last branch, we found that GERD (nodes 83&84) is the next best predictor in patients who did not have respiratory failure, did not receive invasive or non-invasive MV, were non-smokers, had HP, and their age (75.5–90.5). In this subgroup, GERD didn’t predict survival. If patients did not have GERD (Node 83), we predicted that they would survive 90.3% of the time. This rule was applied to 474,863 patients and was accurate 428,913 times. If patients have GERD (Node 84), we predicted that they would survive 94.2% of the time. This rule was applied to 121,608 patients, and we were accurate 114,526 times. Out of the 6 GERD terminal nodes, three GERD terminal nodes (78, 91, 92) that have index > 100% indicating that these nodes have higher observed cases when compared to the expected cases of patients who died. Tree tables are summarized in Additional file 1: Table S9.

The results of the training cohort decision tree are similar to our validation cohort regarding GERD's ranking as a predictor among different ILD patients' subgroups. However, the GERD prediction accuracy was higher for mortality and lower for survival as we oversampled the mortality outcome data to enhance our model parameters.

### Model performance and predictor importance:

After applying our new model (after resampling) to our validation cohort, our model had a sensitivity of 73.43%, specificity of 66.15%, precision of 0.27, NPV of 93.62%, accuracy of 67.2%, MCC of 0.3, F1 score of 0.4, and AUC for the ROC of 0.76 (Table 2). The metrics from the training cohort were similar to the validation cohort, except that the training cohort model performance has higher precision (0.68), MCC (0.4), F1 score (0.7), and lower NPV (71.6%) (Additional file 1: Table S8). GERD importance was calculated as 0.003, and compared to other predictors, the normalized importance was 5% (Additional file 1: Figure S2 and Table S10).

### Additional analysis

The Gain and index charts and tables are depicted in the supplementary material (Additional file 1: Table S11 and Figures S3-4.). Additional file 1: Tables S12-15 depict risk estimates and confusion matrices. Results from the time-based validation cohort CART secondary analysis showed similar results (Additional file 1: Figure S5). The results were also similar among different ILD types except in one subgroup (in HP patients), where GERD was the best predictor in patients who received MV (Additional file 1: Figures S6-10).

## Discussion

This analysis investigated the relationship between GERD and the combined cohort of ILD-related hospitalizations with IPF, CTD-ILD, HP, pulmonary sarcoidosis, and unspecified-ILD. We identified and validated that GERD does not have a prognostic value in ILD-related hospitalizations. Also, GERD is a strongly associated with hospitalized ILD patients who did not receive MV (invasive, non-invasive, or both) irrespective of the respiratory failure diagnosis. Besides, patients were not dependent on LTOT. These findings highlight that GERD might be associated with mild ILD-related hospitalizations. This didn’t change across different ILD subtypes except in HP-related hospitalizations as only one out of five subgroups where GERD was a strong predictor (of mortality?) in patients who received invasive MV. Also, we validated this across time, indicating that the change of health care practice did not impact the GERD association with mild ILD-related hospitalization. To our knowledge, this is the first study that used supervised machine learning in a large dataset to study the impact of GERD on ILD-related hospitalizations.

Although our initial classifier showed an accuracy of 85.5%, it underperformed in other metrics like sensitivity, precision, MCC, and F1 score. Accuracy is very sensitive to our data since it has an imbalanced outcome (14.5% mortality vs. 85.5% survival). This phenomenon is known as accuracy paradox [25, 26]. To improve our model performance, we oversampled the ILD-related hospitalizations for those who died so we could have a balanced outcome. To avoid overfitting, we only oversampled the training cohort, which explains why parameters like F1 and MCC are lower in the validation sample compared to the training set. The F1 tackles data imbalance. In our models, it was 0 before resampling and improved to 0.4. However, the F1 score is asymmetric as it focuses on positive cases only (patients who died). Given the above, we used the MCC. It considers all four values in the confusion matrix. MCC is considered more informative when compared to other metrics for binary classifiers [27].

Our analysis highlights that GERD is not associated with survival for two reasons: (1) MV is a known predictor of mortality in ILD-related hospitalizations [12, 28, 29]. Considering this fact, MV might be a surrogate of respiratory failure severity. Our investigation showed that GERD is strongly associated with ILD-related hospitalizations for patients who did not receive MV irrespective of their respiratory failure status (i.e. mild respiratory failure). (2) Our model performed well in predicting survival whether GERD is present or not in these subgroups (Fig. 2. Nodes: 53&54; 83&84) and performed poorly in predicting mortality whether GERD is present or not (Fig. 2. Nodes: 77&78; 91&92). In node 73, the model performed poorly for those who did not have GERD predicting mortality, while it performed well for those with GERD predicting survival. In our subgroup analysis, these results did not change except in one subgroup of HP patients with which GERD was a strong predictor associated with invasive MV (Additional file 1: Figure S8. Nodes: 49&50).

An underpowered phase 2 trial showed that Nissen fundoplication for IPF treatment did not have a significant impact on lung function decline [30]. The surgery group had less exacerbation, hospitalizations, and death without statistical significance [30]. Wong et al. showed that GERD is one of the most prevalent comorbidities among all ILD [31]. In their cluster-based analysis, GERD was associated with reduced survival in IPF, while it had no impact on other forms of ILD [31]. However, their model was not validated. Multiple retrospective studies showed contradictory results regarding the use of anti-acid treatment in IPF patients [32]. The discrepancies in these results might be explained by immortal time bias [32]. Since the NIS database does not include medication data, our investigation could not address whether anti-acid therapy will impact survival in ILD-related hospitalization, and we could not assess for this bias at the time of admission.

On the other hand, our validated model suggests that mild ILD-related hospitalizations are associated with improved survival irrespective of GERD diagnosis. Due to that, the availability of anti-acid therapy data should not change GERD's role in our model. Keeping in mind that having a GERD diagnosis does not imply that patients receive anti-acid therapy. Other studies showed that GERD is associated with higher survival in pulmonary sarcoidosis [12] and but not in Chronic HP [33].

We investigated the role of GERD in a combined ILD cohort since different ILD might share a progressive fibrotic phenotype [34–36]. Different studies suggested that ILD with progressive phenotype might share underlying molecular mechanisms [34, 36]. We also performed a subgroup analysis (by ILD subtype) since we could not identify ILD hospitalizations with progressive fibrotic phenotype. Our subgroup analysis showed that GERD does not have a prognostic value in our validated models across different ILD subtypes. Although GERD might play a different role in different ILD subtypes, its prognostic value has been consistent.

Our study has several limitations. First, NIS data identifies hospitalizations, not unique patients. As a result, patients with multiple admissions might be overrepresented. Our model reflects ILD-related hospitalizations predictors rather than patients with ILD. To mitigate this problem, we performed time-based data splitting, which would decrease the overlap of repeated hospitalizations for ILD patients. Second, miscoding bias is expected since the NIS database entirely depends on ICD-9-CM and ICD-10-CM coding. For example, our data showed patients who were not diagnosed with respiratory failure but received MV. This can be explained by either miscoding or MV for elective procedures. To minimize the problem of ILD miscoding, we included cases with multiple ILD diagnoses in our analysis and subgroup analysis. Third, the details on how GERD was diagnosed are not available. Fourth,  NIS database does not have baseline pulmonary function tests, radiographic patterns, medications (immunosuppression or antifibrotic) used to treat ILD patients during hospitalization, and the primary diagnosis for admission (if it is related to respiratory failure or not). Finally, mechanical ventilation would complicate the results for patients with IPF since it is not recommended. The results of the IPF cohort analysis should be interpreted cautiously.

## Conclusion

We developed and validated a model using a supervised method that investigates the prognostic role of GERD in a combined cohort of ILD-related hospitalizations. Our CART model suggested that diagnosis of GERD is associated with mild ILD-related hospitalization but did not predict survival compared to patients who did not have GERD. Our study does not address whether anti-acid therapy impacts ILD-related hospitalization. We cannot extend these results to the outpatient world. Further studies are needed to evaluate the relationship between GERD and ILD in the outpatient world.

## Supplementary Information

**Additional file 1.** Supplementary Materials.

## Data Availability

The data that support the findings of this study are available on request from the corresponding author, [S.A.]. The data are publicly available: https://www.hcup-us.ahrq.gov/nisoverview.jsp.

## Abbreviations

GERD: Gastroesophageal reflux disease
ILD: Interstitial lung disease
NIS: National Inpatient Sample
CART: Classification and regression tree
IPF: Idiopathic pulmonary fibrosis
CTD-ILD: Connective tissue diseases–ILD
HP: Hypersensitivity pneumonitis
RA-ILD: Rheumatoid arthritis-ILD
DMPM-ILD: Dermatomyositis/polymyositis-ILD
SSc-ILD: Scleroderma-ILD
RCT: Randomized controlled trials
SML: Supervised machine learning
AHRQ: Agency for Healthcare Research and Quality
ICD: International Classification of Diseases
COPD: Chronic obstructive pulmonary disease
OSA: Obstructive sleep apnea
HH: Hiatal hernia
LBMI: Low body mass index
pHTN: Pulmonary hypertension
PE: Acute pulmonary embolism
LTOT: Long term oxygen therapy
MV: Mechanical ventilation
