# Supplementary material for: The prognostic value of gastroesophageal reflux disorder in interstitial lung disease related hospitalizations
Source: Respir Res. 2023 Mar 30;24:97. doi: 10.1186/s12931-023-02407-4 (PMC10061884; doi:10.1186/s12931-023-02407-4)
Supplement: Supplementary file 1 — Additional file 1. Supplementary Materials. [file 12931_2023_2407_MOESM1_ESM.docx]

| Autoimmune syndrome codes | | | | | | |
| --- | --- | --- | --- | --- | --- | --- |
|  |  | ICD 10 |  |  | ICD9 |  |
| 1. Rheumatoid Arthritis | M05.XX | M06.XX |  | 714.0 | 714.1 | 714.2 |
|  |  |  |  | 714.81 | 714.89 | 714.9 |
| 1. Scleroderma | M34.XX |  |  | 710.1 |  |  |
| 1. Dermatomyositis and Polymyositis | M33.XX |  |  | 710.3 | 710.4 |  |
| Specific CTD-ILD codes | | | | | | |
| 1. RA-ILD specific codes | M05.1X |  |  | 714.81 |  |  |
| 1. DMPM-ILD specific codes | M33.11 | M33.21 | M33.91 |  |  |  |
| 1. SSc-ILD specific codes | M34.81 |  |  | 517.2 |  |  |
| Other pulmonary involvement codes | | | | | | |
| 1. Post-inflammatory fibrosis | J84.10 |  |  | 515 |  |  |
| 1. Nonspecific pulmonary fibrosis | J84.111 | J84.89 |  | 516.30 |  |  |
| 1. Lung involvement in other diseases classified elsewhere |  |  |  | 517.8 |  |  |

**Supplementary Materials**

- RA-ILD is defined as (4) **OR** (1) and (7) **OR** (1) and (8).
- DMPM-ILD is defined as (5) **OR** (2) and (7) **OR** (2) and (8) **OR** (2) and (9).
- SSc-ILD is defined as (6) **OR** (3) and (7) **OR** (3) and (8).
- CTD-ILD: any RA-ILD, DMPM-ILD, SSc-ILD or more than one of these diagnoses.

**Table E1**: selection Criteria for CTD-ILD

| **Diagnoses** | **ICD 10 codes** | | | **ICD 9 codes** | | |
| --- | --- | --- | --- | --- | --- | --- |
| Gastroesophageal reflux disease | K21.0 | K21.9 |  | 530.11 | 530.81 |  |
| Barret’s esophagus | K22.70 | K22.710 | K22.711 | 530.85 |  |  |
|  | K22.719 |  |  |  |  |  |
| Hiatal hernia | K44.0 | K44.1 | K44.9 | 551.3 | 552.3 | 553.3 |
| Ever smoker: |  |  |  |  |  |  |
| Current | F17.200 | F17.201 | F17.203 | 305.1 |  |  |
|  | F17.208 | F17.209 | F17.210 |  |  |  |
|  | F17.211 | F17.213 | F17.218 |  |  |  |
|  | F1.7219 |  |  |  |  |  |
| Previous | Z87.891 |  |  | V15.82 |  |  |
| Dependence on long-term oxygen | Z99.81 |  |  | V46.2 |  |  |
| Respiratory failure (acute, acute on chronic, chronic, unspecified) | J96.00 | J96.01 | J96.02 | 518.81 |  |  |
|  | J96.20 | J96.21 | J96.22 | 518.84 |  |  |
|  | J96.10 | J96.11 | J96.12 | 518.83 |  |  |
|  | J96.90 | J96.91 | J96.92 |  |  |  |
| Chronic obstructive lung disease | J44.0 | J44.1 | J44.9 | 491.20 | 491.21 | 491.22 |
| Obstructive sleep apnea | G47.33 |  |  | 327.23 |  |  |
| Frailty | R54 |  |  | 797 |  |  |
| Low body mass index (<20) | Z68.1 |  |  | V85.0 |  |  |
| Acute pulmonary embolism | I26.01 | I26.02 | I26.09 | 415.11 | 415.12 | 415.13 |
|  | I26.90 | I26.92 | I26.93 | 415.19 |  |  |
|  | I26.94 | I26.99 |  |  |  |  |
| Pulmonary Hypertension | I270 | I27.2X | I27.89 | 416.0 | 416.8 | 416.9 |
| Any history of lung transplant | Z94.2 | Z94.3 | Z48.24 | V42.6 |  |  |
|  | Z48.280 |  |  |  |  |  |
| **Procedures** | | | | | | |
| Invasive mechanical ventilation | 5A1935Z | 5A1945Z | 5A1955Z | 96.7 | 96.70 | 96.71 |
|  |  |  |  | 96.72 |  |  |
| Non-invasive mechanical ventilation | 5A09357 | 5A09457 | 5A09557 | 93.91 | 93.90 |  |
| Lung transplant | 0BYC0Z0 | 0BYC0Z1 | 0BYC0Z2 | 33.50 | 33.51 | 33.52 |
|  | 0BYD0Z0 | 0BYD0Z1 | 0BYD0Z2 | 33.6 |  |  |
|  | 0BYF0Z0 | 0BYF0Z1 | 0BYF0Z2 |  |  |  |
|  | 0BYG0Z0 | 0BYG0Z1 | 0BYG0Z2 |  |  |  |
|  | 0BYH0Z0 | 0BYH0Z1 | 0BYH0Z2 |  |  |  |
|  | 0BYJ0Z0 | 0BYJ0Z1 | 0BYJ0Z2 |  |  |  |
|  | 0BYK0Z0 | 0BYK0Z1 | 0BYK0Z2 |  |  |  |
|  | 0BYL0Z0 | 0BYL0Z1 | 0BYL0Z2 |  |  |  |
|  | 0BYM0Z0 | 0BYM0Z1 | 0BYM0Z2 |  |  |  |

**Table E2.** International Classification of Diseases (ICD) codes for ninth and tenth editions that are used in this study.

|  | **Clinical Classification System Refined (CCSR) for ICD-10 codes** | **Clinical Classification Software for ICD-9 codes** |
| --- | --- | --- |
| Pneumonia | RSP002 | 122 |
| Asthma | RSP009 | 128 |
| **Procedures:** |  | |
| Diagnostic bronchoscopy and biopsy of bronchus | 37 | |

**Table E3** Procedures and diagnoses categories extracted using clinical classification system tools for ICD-9 and ICD-10 (for procedures and diagnoses).^1-3^

The Clinical Classifications Software Refined (CCSR) for ICD-10-CM and the Clinical Classifications Software (CCS) for ICD-9-CM are software that categorize ICD diagnosis codes into a manageable number of clinically meaningful categories. It aggregates over 70,000 ICD-10-CM diagnosis codes and over 14,000 ICD-9-CM diagnosis codes. CCS-Services and Procedures provides a method for classifying Current Procedural Terminology (CPT®) codes and Healthcare Common Procedure Coding System (HCPCS) codes into clinically meaningful procedure categories. It aggregates more than 9,000 CPT/HCPCS codes and 6,000 HCPCS codes are collapsed into 244 clinically meaningful categories.^1^ Obesity was abstracted using Elixhauser Comorbidity Software.^2,3^

**Supervised machine learning approach**

Univariable logistic regression for variables selection

Creating training and validation sets

Training cohort (60%)

Validation cohort (40%)

CART analysis

Resampled training cohort

Model evaluation^*^

Model evaluation^*^

CART analysis

Validation cohort (unchanged)

Bootstrap-based technique was used to resample the imbalanced data

*Model metrics: sensitivity, specificity, precision, F1 score, accuracy rate, risk estimation and ROC curve

**Figure E1:** Statistical analysis approach. (1) univariable logistic regression of the variables: age, gender, race, smoking history, ILD subtype, low BMI, obesity, GERD, OSA, frailty, RF, use of MV, bronchoscopy, urban vs. rural hospital location, academic hospital status, COPD, asthma, acute PE, pHTN, HH, Barrett’s esophagus, pneumonia, and dependence on long-term oxygen. Variables whose p-value < 0.05 were selected to be included in the CART analysis; (2) The data is randomly split into training set (60%) and validation set (40%); (3) Models’ metrics that are used for model evaluation: sensitivity, specificity, precision, F1 score, accuracy rate, risk estimation and ROC curve; (4) Resampled the training data set (using bootstrap-based technique) to manage the imbalanced outcome and enhance out model performance; (5) CART re-analysis, and evaluation of the new model use the same metrics;(6)&(7) testing the new model from the resampled training dataset for subgroup analysis (divided by the ILD type) and time based (Training data set from years 2011-2013 and the validation set is from (2016-2019).

**Missing value analysis:**

|  | Counts | % |
| --- | --- | --- |
| Mortality (died) | 6897 | 0·088 |
| Age | 1704 | 0·022 |
| Sex | 910 | 0·012 |
| Race | 570913 | 7·31 |
| Length of hospital stay | 1247 | 0·016 |
| Urban hospitals | 22245 | 0·285 |
| Academic hospitals | 22245 | 0·285 |
| Bed size of hospital | 22245 | 0·285 |

**Table E4**. List of variables with missing values (count and percentage). All are below < 10%. No imputation required.

**Unadjusted logistic regression:**

|  | **OR** | **95% CI** | **p-value** |
| --- | --- | --- | --- |
| **Age** | 1·017 | (1·0169- 1·0172) | <0·001 |
| **Female** | 1·006 | (1·002- 1·009) | <0·001 |
| **Race*** |  | | |
| **Black** | 0·95 | (0·945- 0·956) | <0·001 |
| **Hispanic** | 1·067 | (1·059- 1·075) | <0·001 |
| **Asian or Pacific Islander** | 1·419 | (1·403- 1·434) | <0·001 |
| **Native American** | 1·05 | (1·023- 1·078) | <0·001 |
| **Other** | 1·182 | (1·168- 1·196) | <0·001 |
| **Eversmoker** | 0·646 | (0·643- 0·649) | <0·001 |
| **Interstitial lung disease subtype:** |  | | |
| **Idiopathic pulmonary fibrosis** | 0·674 | (0·664- 0·684) | <0·001 |
| **Connective tissue disease-ILD** | 0·358 | (0·353- 0·363) | <0·001 |
| **Hypersensitivity pneumonitis** | 2·757 | (2·731- 2·783) | <0·001 |
| **Pulmonary sarcoidosis** | 0·137 | (0·134- 0·141) | <0·001 |
| **Unspecified-ILD** | 1·096 | (1·064- 1·129) | <0·001 |
| **Co-morbidities** |  | | |
| **Gastroesophageal reflux disease** | 0·588 | (0·585- 0·591) | <0·001 |
| **Respiratory failure** | 3·861 | (3·845- 3·878) | <0·001 |
| **Dependence on long-term Oxygen** | 0·658 | (0·652- 0·664) | <0·001 |
| **Chronic obstructive lung disease** | 0·748 | (0·743- 0·753) | <0·001 |
| **Asthma** | 0·56 | (0·554- 0·565) | <0·001 |
| **Pneumonia** | 1·115 | (1·109- 1·12) | <0·001 |
| **Acute pulmonary embolism** | 1·652 | (1·632- 1·672) | <0·001 |
| **Pulmonary hypertension** | 0·99 | (0·982- 0·998) | 0·02 |
| **Obstructive sleep apnea** | 0·582 | (0·577- 0·588) | <0·001 |
| **Obesity** | 0·773 | (0·768- 0·779) | <0·001 |
| **Barrett's esophagus** | 0·536 | (0·515- 0·557) | <0·001 |
| **Hiatal hernia** | 0·551 | (0·544- 0·558) | <0·001 |
| **Low body mass index** | 1·192 | (1·182- 1·202) | <0·001 |
| **Frailty** | 1·175 | (1·134- 1·217) | <0·001 |
| **Bronchoscopy** | 1·445 | (1·435- 1·455) | <0·001 |
| **Invasive mechanical ventilation** | 3·699 | (3·684- 3·714) | <0·001 |
| **Non-invasive mechanical ventilation** | 1·601 | (1·589- 1·613) | <0·001 |
| **Both invasive and non-invasive mechanical ventilation** | 2·471 | (2·445- 2·498) | <0·001 |
| **Academic hospitals** | 1·085 | (1·081- 1·089) | <0·001 |
| **Urban hospitals** | 1·218 | (1·21- 1·226) | <0·001 |

**Table E5**. Unadjusted logistic regression for variables included in CART analysis. *Comparted to white.

**Baseline characteristics for the validation and training sets:**

|  | **ILD patients with GERD (n=701,121)** | **ILD patients without GERD (n=2,728,848)** | **p-value** |
| --- | --- | --- | --- |
| **Age, mean ± SD** | 71·18 (15·24) | 69·51 (17·19) | <0·001 |
| **Age group (20-39), n (%)** | 26537 (4) | 190769 (7) | <0·001 |
| **Age group (40-59), n (%)** | 125752 (18) | 527189 (19) | <0·001 |
| **Age group (60-79), n (%)** | 298949 (43) | 1049400 (38) | <0·001 |
| **Age group (80 or more), n (%)** | 249598 (36) | 961032 (35) | 0·006 |
| **Female, n (%)** | 337228 (48) | 1130513 (41) | <0·001 |
| **Race** |  |  | <0·001 |
| **White, n (%)** | 519866 (79) | 1821204 (72) |  |
| **Black, n (%)** | 72631 (11) | 343007 (14) |  |
| **Hispanic, n (%)** | 36994 (6) | 196472 (8) |  |
| **Asian or Pacific Islander, n (%)** | 11796 (2) | 75166 (3) |  |
| **Native American, n (%)** | 3242 (0) | 15109 (1) |  |
| **Other, n (%)** | 13069 (2) | 69733 (3) |  |
| **Smoking Status** |  |  |  |
| **Ever smoker, n (%)** | 211232 (30) | 679912 (25) | <0·001 |
| **Interstitial lung disease subtype:** |  |  | <0·001 |
| **Idiopathic pulmonary fibrosis, n (%)** | 25266 (4) | 50428 (2) |  |
| **Connective tissue disease-ILD, n (%)** | 41054 (6) | 85513 (3) |  |
| **Hypersensitivity pneumonitis, n (%)** | 603262 (86) | 2504222 (92) |  |
| **Pulmonary sarcoidosis, n (%)** | 26955 (4) | 79559 (3) |  |
| **Unspecified-ILD, n (%)** | 4584 (1) | 9125 (0) |  |
| **Co-morbidities** |  |  |  |
| **Respiratory failure, n (%)** | 300564 (43) | 1319401 (48) | <0·001 |
| **Dependence on long-term Oxygen, n (%)** | 64425 (9) | 140874 (5) | <0·001 |
| **Chronic obstructive lung disease, n (%)** | 85533 (12) | 262787 (10) | <0·001 |
| **Asthma, n (%)** | 67033 (10) | 145920 (5) | <0·001 |
| **Pneumonia, n (%)** | 106657 (15) | 442504 (16) | <0·001 |
| **Acute pulmonary embolism, n (%)** | 11302 (2) | 52626 (2) | <0·001 |
| **Pulmonary hypertension, n (%)** | 47060 (7) | 141170 (5) | <0·001 |
| **Obstructive sleep apnea, n (%)** | 68755 (10) | 151273 (6) | <0·001 |
| **Obesity, n (%)** | 81063 (12) | 244117 (9) | <0·001 |
| **Barrett's esophagus, n (%)** | 6461 (1) | 6595 (0) | <0·001 |
| **Hiatal hernia, n (%)** | 60331 (9) | 65843 (2) | <0·001 |
| **Low body mass index, n (%)** | 31447 (4) | 125939 (5) | 0·038 |
| **Frailty, n (%)** | 2134 (0·3) | 6416 (0·2) | <0·001 |
| **Elixhauser sum of conditions** |  |  |  |
| **Mean ± SD** | 2·31 (2·54) | 2·46 (2·43) | <0·001 |
| **Median (IQR)** | 2(4) | 2(4) |  |
|  |  |  |  |
| **Hospitalization Characteristics** |  |  |  |
| **Length of hospital stay, mean ± SD** | 8·21 (9·00) | 10·39 (12·78) | <0·001 |
| **In-patient pulmonary Procedures** |  |  |  |
| **Bronchoscopy, n (%)** | 40199 (6) | 184847 (7) | <0·001 |
| **Invasive mechanical ventilation, n (%)** | 105998 (15) | 717437 (26) | <0·001 |
| **Non-invasive mechanical ventilation, n (%)** | 38985 (6) | 130805 (5) | <0·001 |
| **Mechanical ventilation (invasive and non-invasive), n (%)** | 10671 (1·5) | 57936 (2·1) | <0·001 |
| **Bed size of hospital** |  |  | <0·001 |
| **Small, n (%)** | 122922 (18) | 451967 (17) |  |
| **Medium, n (%)** | 194687 (28) | 738772 (27) |  |
| **Large, n (%)** | 381805 (55) | 1529766 (56) |  |
| **Region of hospital** |  |  | <0·001 |
| **Northeast, n (%)** | 130144 (19) | 550829 (20) |  |
| **Midwest, n (%)** | 171421 (24) | 568024 (21) |  |
| **South, n (%)** | 273586 (39) | 1022366 (37) |  |
| **West, n (%)** | 125970 (18) | 587628 (22) |  |
| **Academic hospitals, n (%)** | 389961 (56) | 1493358 (55) | <0·001 |
| **Urban hospitals, n (%)** | 617070 (88) | 2440405 (90) | <0·001 |

**Table E6·** Hospitalizations characteristics for ILD patients with GERD vs. without GERD in the validation cohort.

|  | **ILD patients with GERD (n=1,050,070)** | **ILD patients without GERD (n=4,088,693)** | **p-value** |
| --- | --- | --- | --- |
| **Age, mean ± SD** | 71·21 (15·27) | 69·55 (17·19) | <0·001 |
| **Age group (20-39), n (%)** | 40708 (4) | 286605 (7) | <0·001 |
| **Age group (40-59), n (%)** | 188250 (18) | 785917 (19) | <0·001 |
| **Age group (60-79), n (%)** | 444519 (42) | 1571094 (38) | <0·001 |
| **Age group (80 or more), n (%)** | 376270 (36) | 1444277 (35) | <0·001 |
| **Female, n (%)** | 504212 (48) | 1696174 (41) | <0·001 |
| **Race** |  |  | <0·001 |
| **White, n (%)** | 778446 (79) | 2737267 (72) |  |
| **Black, n (%)** | 108263 (11) | 511088 (14) |  |
| **Hispanic, n (%)** | 55272 (6) | 290175 (8) |  |
| **Asian or Pacific Islander, n (%)** | 18398 (2) | 112978 (3) |  |
| **Native American, n (%)** | 4975 (1) | 22629 (1) |  |
| **Other, n (%)** | 19720 (2) | 102701 (3) |  |
| **Smoking Status** |  |  |  |
| **Ever smoker, n (%)** | 317805 (30) | 1018852 (25) | <0·001 |
| **Interstitial lung disease subtype:** |  |  | <0·001 |
| **Idiopathic pulmonary fibrosis, n (%)** | 38491 (4) | 76121 (2) |  |
| **Connective tissue disease-ILD, n (%)** | 61566 (6) | 126045 (3) |  |
| **Hypersensitivity pneumonitis, n (%)** | 903492 (86) | 3754623 (92) |  |
| **Pulmonary sarcoidosis, n (%)** | 39751 (4) | 118456 (3) |  |
| **Unspecified-ILD, n (%)** | 6770 (1) | 13449 (0) |  |
| **Co-morbidities** |  |  |  |
| **Respiratory failure, n (%)** | 449192 (43) | 1974778 (48) | <0·001 |
| **Dependence on long-term Oxygen, n (%)** | 96709 (9) | 212820 (5) | <0·001 |
| **Chronic obstructive lung disease, n (%)** | 125981 (12) | 392863 (10) | <0·001 |
| **Asthma, n (%)** | 99219 (9) | 218653 (5) | <0·001 |
| **Pneumonia, n (%)** | 158545 (15) | 663275 (16) | <0·001 |
| **Acute pulmonary embolism, n (%)** | 16362 (2) | 79206 (2) | <0·001 |
| **Pulmonary hypertension, n (%)** | 70214 (7) | 210119 (5) | <0·001 |
| **Obstructive sleep apnea, n (%)** | 102518 (10) | 226358 (6) | <0·001 |
| **Obesity, n (%)** | 121592 (12) | 368452 (9) | <0·001 |
| **Barrett's esophagus, n (%)** | 9680 (1) | 9859 (0) | <0·001 |
| **Hiatal hernia, n (%)** | 89646 (9) | 99127 (2) | <0·001 |
| **Low body mass index, n (%)** | 47143 (4) | 190113 (5) | 0·002 |
| **Frailty, n (%)** | 3384 (0·3) | 9958 (0·2) | <0·001 |
| **Elixhauser sum of conditions** |  |  |  |
| **Mean ± SD** | 2·31 (2·53) | 2·46 (2·43) | <0·001 |
| **Median (IQR)** | 2(4) | 2(4) |  |
|  |  |  |  |
| **Hospitalization Characteristics** |  |  |  |
| **Length of hospital stay, mean ± SD** | 8·21 (8·71) | 10·42 (12·95) | <0·001 |
| **In-patient pulmonary Procedures** |  |  |  |
| **Bronchoscopy, n (%)** | 59548 (6) | 278699 (7) | <0·001 |
| **Invasive mechanical ventilation, n (%)** | 159901 (15) | 1071596 (26) | <0·001 |
| **Non-invasive mechanical ventilation, n (%)** | 56916 (5) | 198069 (5) | <0·001 |
| **Mechanical ventilation (invasive and non-invasive), n (%)** | 16011 (1·5) | 85690 (2·1) | <0·001 |
| **Bed size of hospital** |  |  | <0·001 |
| **Small, n (%)** | 184340 (18) | 672445 (16) |  |
| **Medium, n (%)** | 289087 (28) | 1108723 (27) |  |
| **Large, n (%)** | 573879 (55) | 2295150 (56) |  |
| **Region of hospital** |  |  | <0·001 |
| **Northeast, n (%)** | 196235 (19) | 822989 (20) |  |
| **Midwest, n (%)** | 255939 (24) | 851075 (21) |  |
| **South, n (%)** | 407642 (39) | 1531269 (37) |  |
| **West, n (%)** | 190254 (18) | 883360 (22) |  |
| **Academic hospitals, n (%)** | 584196 (56) | 2237551 (55) | <0·001 |
| **Urban hospitals, n (%)** | 923409 (88) | 3658184 (90) | <0·001 |

**Table E7.** Hospitalizations characteristics for ILD patients with GERD vs. without GERD in the training cohort.

**Model performance for the training data sets:**

|  | **Original data model (training set)** | **Resampled data model (training set)** |
| --- | --- | --- |
| **Sensitivity (%)** | 0% | 73·83% |
| **Specificity (%)** | 100% | 65·91% |
| **Precision** | N/A | 0·6838 |
| **Negative Predictive Value (%)** | 85·50% | 71·62% |
| **Accuracy (%)** | 85·50% | 69·87% |
| **F1 Score** | 0 | 0·7 |
| **MCC** | N/A | 0·4 |
| **AUC(ROC)** | 0·75 | 0·76 |

**Table E8.** Model metrics from the training cohort before and after resampling. F1 score =(2*precision*recall)/(precision + recall). MCC= TP*TN - FP*FN/sqrt((TP+FP)*(TP+FN)*(TN+FP)*(TN+FN))

**Tree tables for the validation cohort**

| **Node** | **Alive (n)** | **Alive (%)** | **Died (n)** | **Died (%)** | **Total (n)** | **Total (%)** | **Predicted Category** | **Parent Node** | **Variable** | **Improvement** | **Split Values** |
| --- | --- | --- | --- | --- | --- | --- | --- | --- | --- | --- | --- |
| 0 | 2932796 | 85·50% | 497179 | 14·50% | 3429975 | 100·00% | Alive |  |  |  |  |
| 1 | 1250115 | 77·20% | 369852 | 22·80% | 1619968 | 47·20% | Died | 0 | Respiratory failure | 0·05137 | Yes |
| 2 | 1682680 | 93·00% | 127327 | 7·00% | 1810007 | 52·80% | Alive | 0 | Respiratory failure | 0·05137 | No |
| 3 | 515813 | 70·90% | 212141 | 29·10% | 727955 | 21·20% | Died | 1 | Invasive MV | 0·00678 | Yes |
| 4 | 734302 | 82·30% | 157711 | 17·70% | 892013 | 26·00% | Died | 1 | Invasive MV | 0·00678 | No |
| 5 | 68927 | 72·20% | 26556 | 27·80% | 95483 | 2·80% | Died | 2 | Invasive MV | 0·0129 | Yes |
| 6 | 1613753 | 94·10% | 100770 | 5·90% | 1714524 | 50·00% | Alive | 2 | Invasive MV | 0·0129 | No |
| 7 | 236739 | 79·50% | 60938 | 20·50% | 297677 | 8·70% | Died | 3 | Age in years at admission | 0·00327 | <= 60·5 |
| 8 | 279074 | 64·90% | 151203 | 35·10% | 430278 | 12·50% | Died | 3 | Age in years at admission | 0·00327 | > 60·5 |
| 9 | 356910 | 88·10% | 48181 | 11·90% | 405091 | 11·80% | Alive | 4 | Age in years at admission | 0·00469 | <= 73·5 |
| 10 | 377392 | 77·50% | 109530 | 22·50% | 486922 | 14·20% | Died | 4 | Age in years at admission | 0·00469 | > 73·5 |
| 11 | 36215 | 80·80% | 8606 | 19·20% | 44821 | 1·30% | Died | 5 | Age in years at admission | 0·00068 | <= 61·5 |
| 12 | 32712 | 64·60% | 17950 | 35·40% | 50662 | 1·50% | Died | 5 | Age in years at admission | 0·00068 | > 61·5 |
| 13 | 819860 | 96·80% | 26857 | 3·20% | 846717 | 24·70% | Alive | 6 | Age in years at admission | 0·0067 | <= 75·5 |
| 14 | 793894 | 91·50% | 73913 | 8·50% | 867807 | 25·30% | Alive | 6 | Age in years at admission | 0·0067 | > 75·5 |
| 15 | 71393 | 85·70% | 11912 | 14·30% | 83305 | 2·40% | Died | 7 | Age in years at admission | 0·00065 | <= 40·5 |
| 16 | 165346 | 77·10% | 49026 | 22·90% | 214372 | 6·20% | Died | 7 | Age in years at admission | 0·00065 | > 40·5 |
| 17 | 159901 | 69·20% | 71224 | 30·80% | 231125 | 6·70% | Died | 8 | Age in years at admission | 0·00053 | <= 75·5 |
| 18 | 119173 | 59·80% | 79980 | 40·20% | 199153 | 5·80% | Died | 8 | Age in years at admission | 0·00053 | > 75·5 |
| 19 | 326896 | 89·30% | 39224 | 10·70% | 366119 | 10·70% | Alive | 9 | Invasive and Non-invasive MV | 0·00127 | No |
| 20 | 30014 | 77·00% | 8958 | 23·00% | 38971 | 1·10% | Died | 9 | Invasive and Non-invasive MV | 0·00127 | Yes |
| 21 | 324543 | 76·10% | 102033 | 23·90% | 426576 | 12·40% | Died | 10 | Depenedence on long-term O2 | 0·00127 | No |
| 22 | 52849 | 87·60% | 7497 | 12·40% | 60346 | 1·80% | Alive | 10 | Depenedence on long-term O2 | 0·00127 | Yes |
| 23 | 16437 | 85·90% | 2697 | 14·10% | 19134 | 0·60% | Alive | 11 | Age in years at admission | 0·00013 | <= 45·5 |
| 24 | 19778 | 77·00% | 5909 | 23·00% | 25687 | 0·70% | Died | 11 | Age in years at admission | 0·00013 | > 45·5 |
| 25 | 669797 | 96·30% | 25622 | 3·70% | 695419 | 20·30% | Alive | 13 | HP | 0·0009 | Yes |
| 26 | 150062 | 99·20% | 1236 | 0·80% | 151298 | 4·40% | Alive | 13 | HP | 0·0009 | No |
| 27 | 738512 | 91·00% | 72702 | 9·00% | 811214 | 23·70% | Alive | 14 | HP | 0·00114 | Yes |
| 28 | 55382 | 97·90% | 1211 | 2·10% | 56593 | 1·60% | Alive | 14 | HP | 0·00114 | No |
| 29 | 111188 | 75·40% | 36230 | 24·60% | 147417 | 4·30% | Died | 16 | Smoker | 0·00016 | No |
| 30 | 54158 | 80·90% | 12797 | 19·10% | 66955 | 2·00% | Died | 16 | Smoker | 0·00016 | Yes |
| 31 | 104485 | 93·00% | 7905 | 7·00% | 112390 | 3·30% | Alive | 19 | Age in years at admission | 0·00083 | <= 55·5 |
| 32 | 222411 | 87·70% | 31318 | 12·30% | 253729 | 7·40% | Alive | 19 | Age in years at admission | 0·00083 | > 55·5 |
| 33 | 15442 | 81·60% | 3475 | 18·40% | 18917 | 0·60% | Died | 20 | Age in years at admission | 0·00015 | <= 60·5 |
| 34 | 14572 | 72·70% | 5482 | 27·30% | 20054 | 0·60% | Died | 20 | Age in years at admission | 0·00015 | > 60·5 |
| 35 | 310772 | 77·00% | 93006 | 23·00% | 403778 | 11·80% | Died | 21 | Invasive and Non-invasive MV | 0·00055 | No |
| 36 | 13771 | 60·40% | 9027 | 39·60% | 22799 | 0·70% | Died | 21 | Invasive and Non-invasive MV | 0·00055 | Yes |
| 37 | 51761 | 88·30% | 6842 | 11·70% | 58604 | 1·70% | Alive | 22 | Invasive and Non-invasive MV | 0·00021 | No |
| 38 | 1088 | 62·40% | 654 | 37·60% | 1742 | 0·10% | Died | 22 | Invasive and Non-invasive MV | 0·00021 | Yes |
| 39 | 261234 | 97·80% | 5897 | 2·20% | 267131 | 7·80% | Alive | 25 | Age in years at admission | 0·00081 | <= 57·5 |
| 40 | 408563 | 95·40% | 19724 | 4·60% | 428288 | 12·50% | Alive | 25 | Age in years at admission | 0·00081 | > 57·5 |
| 41 | 603238 | 90·40% | 63990 | 9·60% | 667228 | 19·50% | Alive | 27 | Smoker | 0·00076 | No |
| 42 | 135274 | 93·90% | 8712 | 6·10% | 143986 | 4·20% | Alive | 27 | Smoker | 0·00076 | Yes |
| 43 | 68141 | 91·80% | 6076 | 8·20% | 74217 | 2·20% | Alive | 31 | Smoker | 0·00021 | No |
| 44 | 36344 | 95·20% | 1830 | 4·80% | 38173 | 1·10% | Alive | 31 | Smoker | 0·00021 | Yes |
| 45 | 192178 | 86·80% | 29301 | 13·20% | 221479 | 6·50% | Alive | 32 | OSA | 0·00052 | No |
| 46 | 30232 | 93·70% | 2018 | 6·30% | 32250 | 0·90% | Alive | 32 | OSA | 0·00052 | Yes |
| 47 | 261396 | 75·70% | 84053 | 24·30% | 345449 | 10·10% | Died | 35 | COPD | 0·00057 | No |
| 48 | 49376 | 84·70% | 8953 | 15·30% | 58329 | 1·70% | Died | 35 | COPD | 0·00057 | Yes |
| 49 | 43558 | 90·30% | 4682 | 9·70% | 48240 | 1·40% | Alive | 37 | Non-invasive MV | 0·00025 | No |
| 50 | 8203 | 79·20% | 2160 | 20·80% | 10363 | 0·30% | Died | 37 | Non-invasive MV | 0·00025 | Yes |
| 51 | 129155 | 98·70% | 1744 | 1·30% | 130899 | 3·80% | Alive | 39 | Age in years at admission | 0·00012 | <= 47·5 |
| 52 | 132079 | 97·00% | 4153 | 3·00% | 136232 | 4·00% | Alive | 39 | Age in years at admission | 0·00012 | > 47·5 |
| 53 | 308061 | 94·80% | 17060 | 5·20% | 325122 | 9·50% | Alive | 40 | GERD | 0·00042 | No |
| 54 | 100502 | 97·40% | 2664 | 2·60% | 103166 | 3·00% | Alive | 40 | GERD | 0·00042 | Yes |
| 55 | 593725 | 90·70% | 61101 | 9·30% | 654826 | 19·10% | Alive | 41 | Non-invasive MV | 0·0006 | No |
| 56 | 9513 | 76·70% | 2890 | 23·30% | 12402 | 0·40% | Died | 41 | Non-invasive MV | 0·0006 | Yes |
| 57 | 132840 | 94·10% | 8263 | 5·90% | 141103 | 4·10% | Alive | 42 | Non-invasive MV | 0·00013 | No |
| 58 | 2434 | 84·40% | 448 | 15·60% | 2883 | 0·10% | Died | 42 | Non-invasive MV | 0·00013 | Yes |
| 59 | 64184 | 91·50% | 5958 | 8·50% | 70142 | 2·00% | Alive | 43 | Pulmonary Sarcoidosis | 0·00012 | No |
| 60 | 3957 | 97·10% | 118 | 2·90% | 4075 | 0·10% | Alive | 43 | Pulmonary Sarcoidosis | 0·00012 | Yes |
| 61 | 161104 | 85·70% | 26948 | 14·30% | 188052 | 5·50% | Alive | 45 | Depenedence on long-term O2 | 0·00043 | No |
| 62 | 31075 | 93·00% | 2353 | 7·00% | 33427 | 1·00% | Alive | 45 | Depenedence on long-term O2 | 0·00043 | Yes |
| 63 | 122096 | 78·80% | 32794 | 21·20% | 154890 | 4·50% | Died | 47 | Age in years at admission | 0·00035 | <= 83·5 |
| 64 | 139299 | 73·10% | 51259 | 26·90% | 190559 | 5·60% | Died | 47 | Age in years at admission | 0·00035 | > 83·5 |
| 65 | 40226 | 86·10% | 6485 | 13·90% | 46711 | 1·40% | Alive | 48 | Non-invasive MV | 0·00014 | No |
| 66 | 9150 | 78·80% | 2468 | 21·20% | 11618 | 0·30% | Died | 48 | Non-invasive MV | 0·00014 | Yes |
| 67 | 213725 | 94·00% | 13545 | 6·00% | 227270 | 6·60% | Alive | 53 | Smoker | 0·00023 | No |
| 68 | 94336 | 96·40% | 3515 | 3·60% | 97851 | 2·90% | Alive | 53 | Smoker | 0·00023 | Yes |
| 69 | 543439 | 91·10% | 53031 | 8·90% | 596470 | 17·40% | Alive | 55 | Age in years at admission | 0·0005 | <= 90·5 |
| 70 | 50286 | 86·20% | 8070 | 13·80% | 58356 | 1·70% | Alive | 55 | Age in years at admission | 0·0005 | > 90·5 |
| 71 | 28629 | 94·20% | 1755 | 5·80% | 30383 | 0·90% | Alive | 59 | Age in years at admission | 0·00014 | <= 43·5 |
| 72 | 35555 | 89·40% | 4203 | 10·60% | 39759 | 1·20% | Alive | 59 | Age in years at admission | 0·00014 | > 43·5 |
| 73 | 122268 | 84·30% | 22730 | 15·70% | 144998 | 4·20% | Died | 61 | GERD | 0·00033 | No |
| 74 | 38836 | 90·20% | 4218 | 9·80% | 43054 | 1·30% | Alive | 61 | GERD | 0·00033 | Yes |
| 75 | 26449 | 94·40% | 1568 | 5·60% | 28017 | 0·80% | Alive | 62 | Non-invasive MV | 0·00018 | No |
| 76 | 4626 | 85·50% | 785 | 14·50% | 5410 | 0·20% | Died | 62 | Non-invasive MV | 0·00018 | Yes |
| 77 | 93201 | 77·30% | 27419 | 22·70% | 120620 | 3·50% | Died | 63 | GERD | 0·00021 | No |
| 78 | 28895 | 84·30% | 5375 | 15·70% | 34270 | 1·00% | Died | 63 | GERD | 0·00021 | Yes |
| 79 | 118302 | 74·50% | 40570 | 25·50% | 158872 | 4·60% | Died | 64 | Non-invasive MV | 0·0002 | No |
| 80 | 20998 | 66·30% | 10689 | 33·70% | 31686 | 0·90% | Died | 64 | Non-invasive MV | 0·0002 | Yes |
| 81 | 209578 | 94·20% | 12914 | 5·80% | 222492 | 6·50% | Alive | 67 | Non-invasive MV | 0·00015 | No |
| 82 | 4146 | 86·80% | 631 | 13·20% | 4778 | 0·10% | Died | 67 | Non-invasive MV | 0·00015 | Yes |
| 83 | 428913 | 90·30% | 45950 | 9·70% | 474863 | 13·80% | Alive | 69 | GERD | 0·00043 | No |
| 84 | 114526 | 94·20% | 7082 | 5·80% | 121608 | 3·50% | Alive | 69 | GERD | 0·00043 | Yes |
| 85 | 81666 | 82·70% | 17050 | 17·30% | 98716 | 2·90% | Died | 73 | Smoker | 0·00023 | No |
| 86 | 40602 | 87·70% | 5680 | 12·30% | 46281 | 1·30% | Alive | 73 | Smoker | 0·00023 | Yes |
| 87 | 33589 | 91·10% | 3265 | 8·90% | 36854 | 1·10% | Alive | 74 | Non-invasive MV | 0·00014 | No |
| 88 | 5247 | 84·60% | 953 | 15·40% | 6200 | 0·20% | Died | 74 | Non-invasive MV | 0·00014 | Yes |
| 89 | 86533 | 76·60% | 26401 | 23·40% | 112934 | 3·30% | Died | 77 | OSA | 0·00014 | No |
| 90 | 6668 | 86·80% | 1018 | 13·20% | 7686 | 0·20% | Alive | 77 | OSA | 0·00014 | Yes |
| 91 | 91415 | 72·90% | 34027 | 27·10% | 125442 | 3·70% | Died | 79 | GERD | 0·00016 | No |
| 92 | 26887 | 80·40% | 6543 | 19·60% | 33430 | 1·00% | Died | 79 | GERD | 0·00016 | Yes |

**Table E9.** The tree table summary for the validation cohort. GERD nodes and their predicted categories are highlighted.

**Figure E2.** Predictor’s importance to the model (calculated using variable sensitivity-based method). GERD is ranked 11^th^ among the 29 variables included in this model. Invasive mechanical ventilation, respiratory failure and age at admission were the top three variables contributed to the most to the predictive power of your model. Admission to academic hospital, gender, and frailty contributed the least to the predictive power of our model.

**Predictors’ importance to the model:**

| **Independent Variable** | **Importance** | **Normalized Importance** |
| --- | --- | --- |
| **Invasive MV** | 0·064 | 100·00% |
| **Respiratory failure** | 0·051 | 80·60% |
| **Age in years at admission** | 0·024 | 38·00% |
| **HP** | 0·016 | 25·20% |
| **Pulmonary Sarcoidosis** | 0·01 | 16·40% |
| **Smoker** | 0·006 | 10·10% |
| **CTD-ILD** | 0·006 | 9·80% |
| **OSA** | 0·006 | 8·80% |
| **Invasive and Non-invasive MV** | 0·004 | 7·00% |
| **Dependence on long-term O2** | 0·004 | 6·50% |
| **GERD** | 0·003 | 5·00% |
| **Obesity** | 0·003 | 4·80% |
| **Asthma** | 0·002 | 3·60% |
| **COPD** | 0·002 | 3·10% |
| **Non-invasive MV** | 0·002 | 2·80% |
| **Hiatal Hernia** | 0·002 | 2·60% |
| **IPF** | 0·001 | 1·30% |
| **Race** | 0·001 | 0·90% |
| **Urban hospital** | 0·001 | 0·80% |
| **Barret's esophagus** | 0·00023 | 0·40% |
| **Bronchoscopy** | 0·00022 | 0·30% |
| **Pneumonia** | 0·00016 | 0·30% |
| **Low BMI** | 0·00011 | 0·20% |
| **Acute PE** | 7·33E-05 | 0·10% |
| **Pulmonary Htn** | 7·15E-05 | 0·10% |
| **Unspecified ILD** | 5·34E-05 | 0·10% |
| **Academic hospital** | 3·94E-05 | 0·10% |
| **Indicator of sex** | 3·47E-06 | 0·0005% |
| **Frailty** | 1·70E-06 | 0·0003% |

**Table E10.** Predictors’ importance to the model (ranked in descending order).

**Gain and index table for the validation cohort:**

| **ID** | **Node N** | **Node %** | **Gain N** | **Gain (%)** | **Response** | **Index** |
| --- | --- | --- | --- | --- | --- | --- |
| 12 | 50662 | 1·50% | 17950 | 3·60% | 35·40% | 244·40% |
| 15 | 83305 | 2·40% | 11912 | 2·40% | 14·30% | 98·60% |
| 17 | 231125 | 6·70% | 71224 | 14·30% | 30·80% | 212·60% |
| 18 | 199153 | 5·80% | 79980 | 16·10% | 40·20% | 277·10% |
| 23 | 19134 | 0·60% | 2697 | 0·50% | 14·10% | 97·20% |
| 24 | 25687 | 0·70% | 5909 | 1·20% | 23·00% | 158·70% |
| 26 | 151298 | 4·40% | 1236 | 0·20% | 0·80% | 5·60% |
| 28 | 56593 | 1·60% | 1211 | 0·20% | 2·10% | 14·80% |
| 29 | 147417 | 4·30% | 36230 | 7·30% | 24·60% | 169·50% |
| 30 | 66955 | 2·00% | 12797 | 2·60% | 19·10% | 131·90% |
| 33 | 18917 | 0·60% | 3475 | 0·70% | 18·40% | 126·70% |
| 34 | 20054 | 0·60% | 5482 | 1·10% | 27·30% | 188·60% |
| 36 | 22799 | 0·70% | 9027 | 1·80% | 39·60% | 273·20% |
| 38 | 1742 | 0·10% | 654 | 0·10% | 37·60% | 259·10% |
| 44 | 38173 | 1·10% | 1830 | 0·40% | 4·80% | 33·10% |
| 46 | 32250 | 0·90% | 2018 | 0·40% | 6·30% | 43·20% |
| 49 | 48240 | 1·40% | 4682 | 0·90% | 9·70% | 67·00% |
| 50 | 10363 | 0·30% | 2160 | 0·40% | 20·80% | 143·80% |
| 51 | 130899 | 3·80% | 1744 | 0·40% | 1·30% | 9·20% |
| 52 | 136232 | 4·00% | 4153 | 0·80% | 3·00% | 21·00% |
| 54 | 103166 | 3·00% | 2664 | 0·50% | 2·60% | 17·80% |
| 56 | 12402 | 0·40% | 2890 | 0·60% | 23·30% | 160·70% |
| 57 | 141103 | 4·10% | 8263 | 1·70% | 5·90% | 40·40% |
| 58 | 2883 | 0·10% | 448 | 0·10% | 15·60% | 107·30% |
| 60 | 4075 | 0·10% | 118 | 0·00% | 2·90% | 19·90% |
| 65 | 46711 | 1·40% | 6485 | 1·30% | 13·90% | 95·80% |
| 66 | 11618 | 0·30% | 2468 | 0·50% | 21·20% | 146·60% |
| 68 | 97851 | 2·90% | 3515 | 0·70% | 3·60% | 24·80% |
| 70 | 58356 | 1·70% | 8070 | 1·60% | 13·80% | 95·40% |
| 71 | 30383 | 0·90% | 1755 | 0·40% | 5·80% | 39·80% |
| 72 | 39759 | 1·20% | 4203 | 0·80% | 10·60% | 72·90% |
| 75 | 28017 | 0·80% | 1568 | 0·30% | 5·60% | 38·60% |
| 76 | 5410 | 0·20% | 785 | 0·20% | 14·50% | 100·00% |
| 78 | 34270 | 1·00% | 5375 | 1·10% | 15·70% | 108·20% |
| 80 | 31686 | 0·90% | 10689 | 2·10% | 33·70% | 232·70% |
| 81 | 222492 | 6·50% | 12914 | 2·60% | 5·80% | 40·00% |
| 82 | 4778 | 0·10% | 631 | 0·10% | 13·20% | 91·20% |
| 83 | 474863 | 13·80% | 45950 | 9·20% | 9·70% | 66·80% |
| 84 | 121608 | 3·50% | 7082 | 1·40% | 5·80% | 40·20% |
| 85 | 98716 | 2·90% | 17050 | 3·40% | 17·30% | 119·20% |
| 86 | 46281 | 1·30% | 5680 | 1·10% | 12·30% | 84·70% |
| 87 | 36854 | 1·10% | 3265 | 0·70% | 8·90% | 61·10% |
| 88 | 6200 | 0·20% | 953 | 0·20% | 15·40% | 106·00% |
| 89 | 112934 | 3·30% | 26401 | 5·30% | 23·40% | 161·30% |
| 90 | 7686 | 0·20% | 1018 | 0·20% | 13·20% | 91·40% |
| 91 | 125442 | 3·70% | 34027 | 6·80% | 27·10% | 187·10% |
| 92 | 33430 | 1·00% | 6543 | 1·30% | 19·60% | 135·00% |

**Table E11.** Terminal nodes gain and index values. Terminal GERD nodes are highlighted. Three GERD terminal nodes (78,91, 92) that have index > 100% indicating that these nodes have higher observed cases when compared to the expected cases of the target population (patient who died).

**
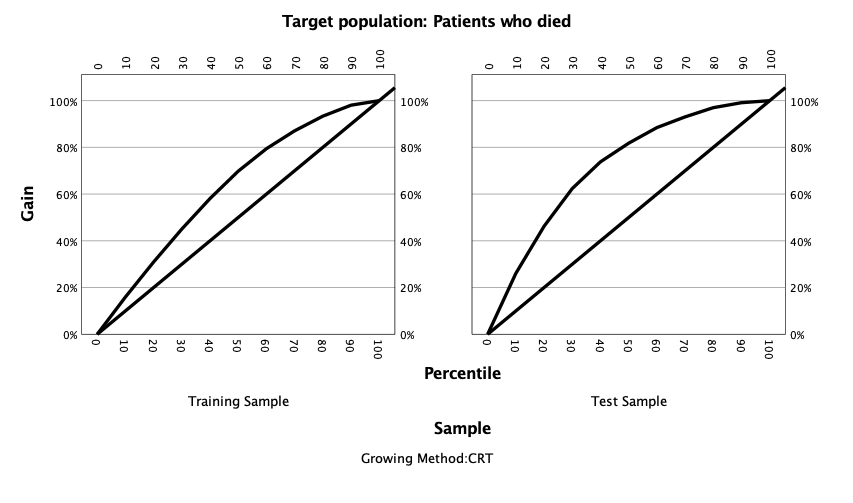
**

**Figure E3.** Gain chart for the training sample (left) and the validation sample (right): The Gain chart for the model applied to the validation cohort indicates that the model is a good model as the gains chart rise steeply toward 100% and then level off. In addition, there is a good separation between the reference line and the model line.

**
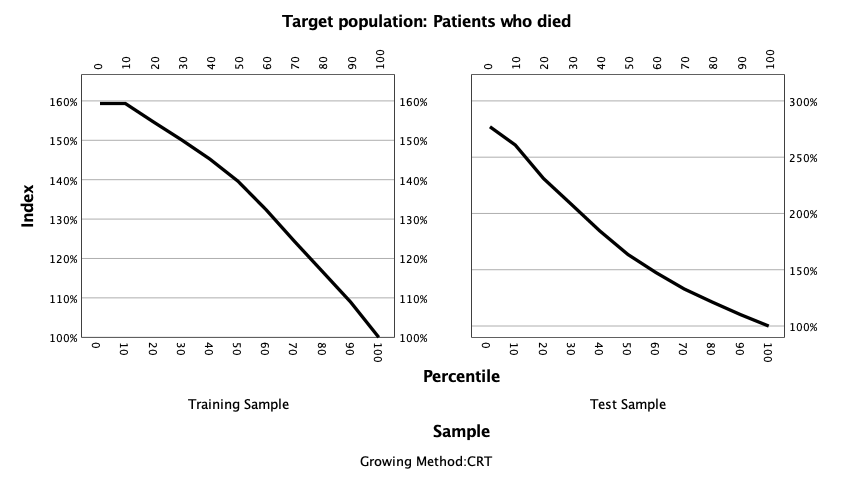
**

**Figure E4.** Index chart for the training sample (left) and the validation sample (right): The Index chart applied to the validation cohort showed that the model provides new information as the cumulative index charts tend to start above 100% and gradually descend until they reach 100%.

**Confusion and risk matrices for training and validation sets before and after training data resampling:**

- **Before resampling:**

| **Sample** | **Estimate** | **Std· Error** |
| --- | --- | --- |
| **Training** | 0·145 | 0·00016 |
| **Validation** | 0·145 | 0·00019 |

**Table E12.** Risk of misclassification of the model in the training and the validation cohort (14·5%)

| **Sample** | **Observed** | **Predicted** | |  |
| --- | --- | --- | --- | --- |
|  |  | **Alive** | **Died** | **Percent Correct** |
| **Training** | Alive | 4404563 | 0 | 100·00% |
|  | Died | 745963 | 0 | 0·00% |
|  | Overall Percentage | 100·00% | 0·00% | 85·50% |
| **Validation** | Alive | 2932796 | 0 | 100·00% |
|  | Died | 497179 | 0 | 0·00% |
|  | Overall Percentage | 100·00% | 0·00% | 85·50% |

**Table E13.** Confusion matrices for the training and the validation cohorts. Overall accuracy is 85·5 in both samples.

- **After resampling:**

| **Sample** | **Estimate** | **Std· Error** |
| --- | --- | --- |
| **Training** | 0·301 | 0·00016 |
| **Validation** | 0·328 | 0·00025 |

**Table E14.** Risk of misclassification of the model in the training (30%) and the validation cohort (32·8%)

| **Sample** | **Observed** | **Predicted** | |  |
| --- | --- | --- | --- | --- |
|  |  | **Alive** | **Died** | **Percent Correct** |
| **Training** | Alive | 2643791 | 1367172 | 65·90% |
|  | Died | 1047731 | 2956338 | 73·80% |
|  | Overall Percentage | 46·10% | 53·90% | 69·90% |
| **Validation** | Alive | 1939906 | 992889 | 66·10% |
|  | Died | 132119 | 365060 | 73·40% |
|  | Overall Percentage | 60·40% | 39·60% | 67·20% |

**Table E15.** Confusion matrices for the training and the validation cohorts. Overall accuracy is 69·9% for the training sample and 67·2% for the validation cohort.


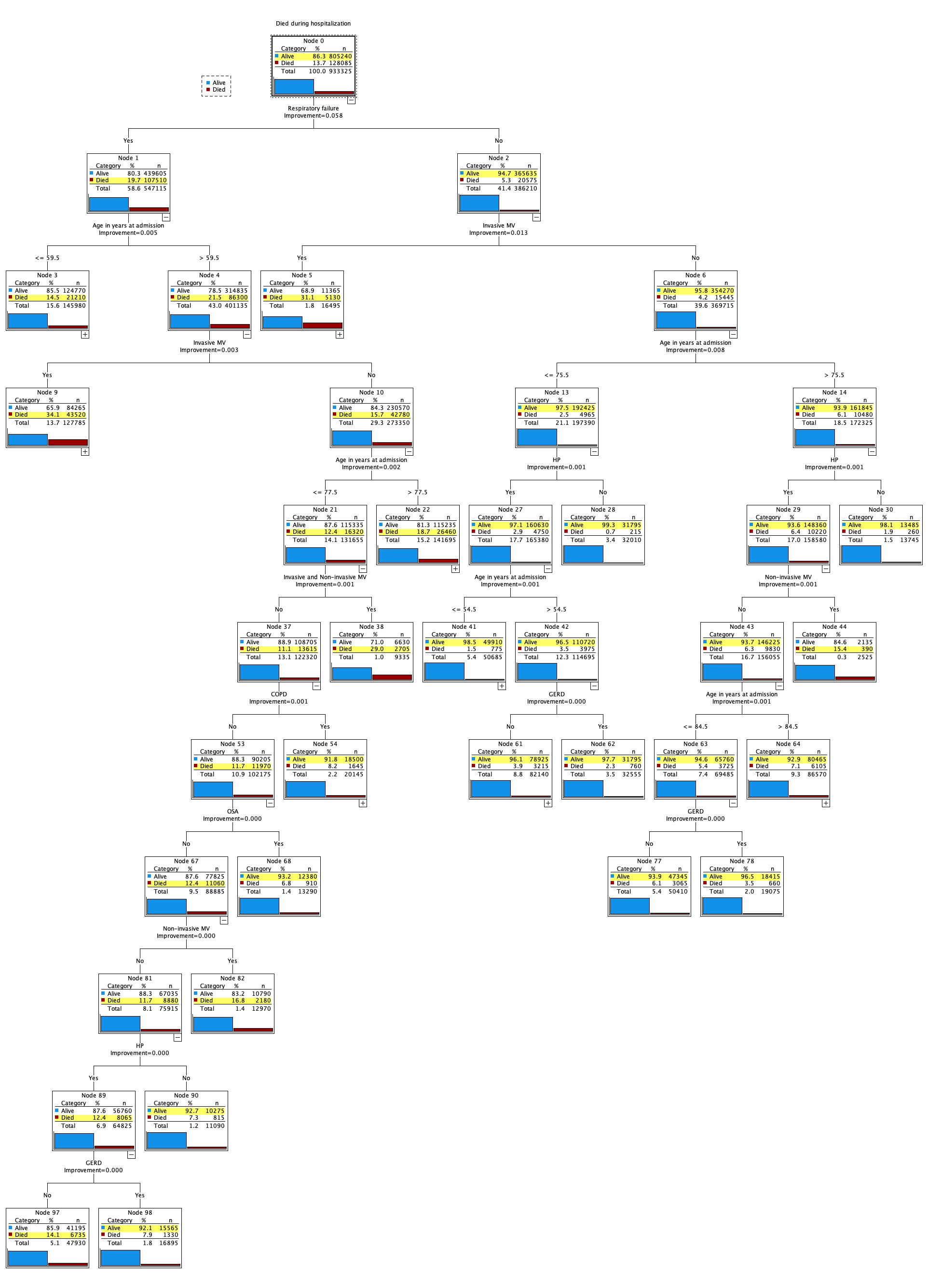
**Decision tree analysis for the time-based validation cohort (2016-2019)**

**Figure E5**. Decision tree for the time-based validation cohort (2016-2019).

In ILD patients hospitalized between 2016 and 2019, GERD (nodes 97& 98) was the best predictor in patients with respiratory failure, didn’t receive any mode of MV, didn’t have OSA or COPD, their age is between 59·5 - 77·5 years and diagnosed with HP. In this subgroup, if patients didn’t have GERD (Node 97), we predicted that they would die 14·1% of the time. This rule was applied to 47930 patients, and we were accurate 41195 times. If patients have GERD (Node 98), we predicted that they survive 92·1% of the time. This rule was applied to 16895 patients, and we were accurate 15565 times. Besides, GERD (nodes 61& 62) was the best predictor in patients without respiratory failure, didn’t receive invasive MV, diagnosed with HP, and their age is between 54·5-75·5 years. In this subgroup, if patients didn’t have GERD (Node 61), we predicted that they survive 96·1% of the time. This rule was applied to 82140 patients, and we were accurate 78925 times. If patients have GERD (Node 62), we predicted that they would survive 97·7% of the time. This rule was applied to 32555 patients, and we were accurate 31795 times. Also, GERD (nodes 77& 78) was the best predictor in patients without respiratory failure, didn’t receive non-invasive MV, diagnosed with HP, their age is between 75·5-85·5 years. In this subgroup, if patients didn’t have GERD (Node 77), we predicted that they would survive 93·9% of the time. This rule was applied to 50410 patients, and we were accurate 47345 times. If patients have GERD (Node 78), we predicted that they would survive 96·5% of the time. This rule was applied to 19075 patients, and we were accurate 18415 times. Results suggest that GERD is the best predictor in patients who did not receive invasive and/or non-invasive mechanical ventilation irrespective of their respiratory failure status

**IPF Decision tree analysis**


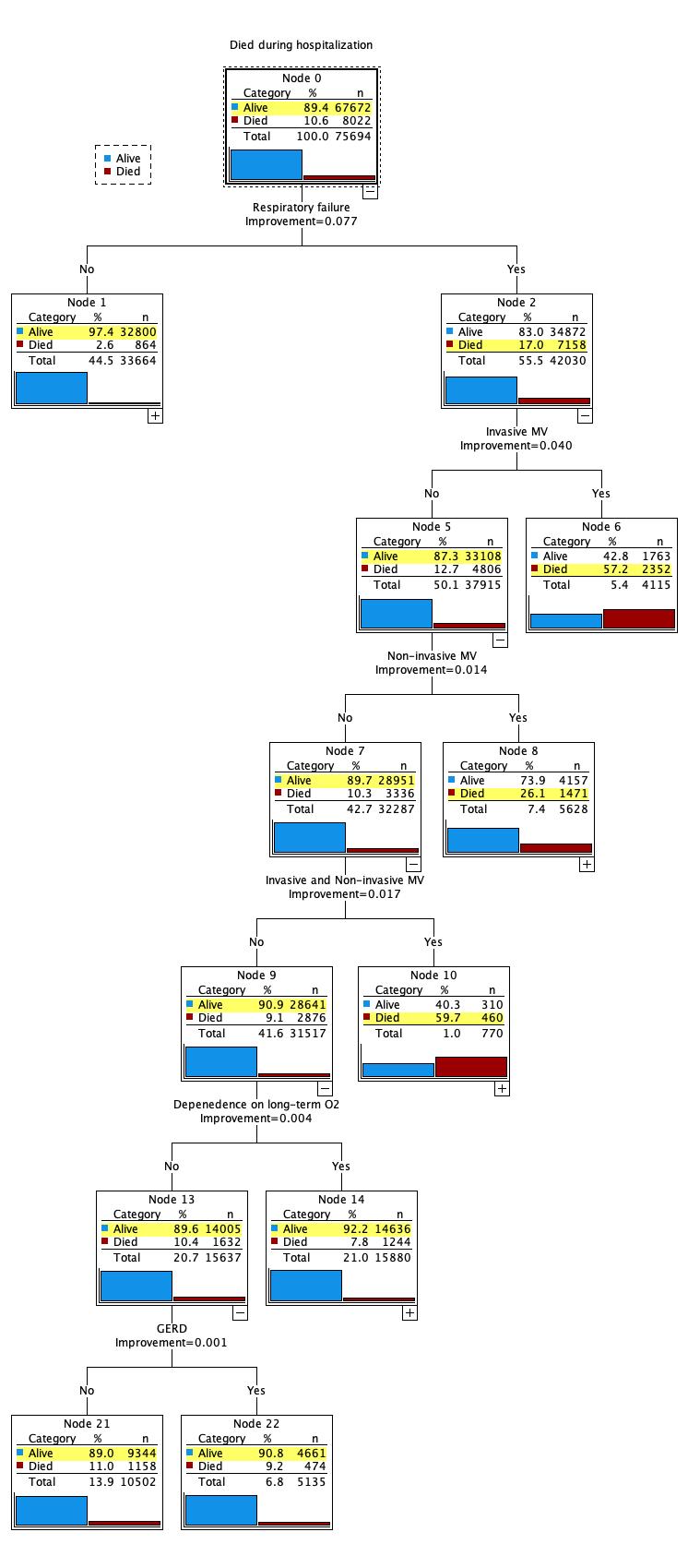


**Figure E6.** Decision tree for IPF patients’ validation cohort.

In IPF patients, GERD (nodes 21& 22) was the best predictor in patients with respiratory failure, didn’t receive any mode of MV and were not on long term oxygen therapy. In this subgroup, if patients didn’t have GERD (Node 21), we predicted that they would survive of 89% of the time. This rule was applied to 10502 patients, and we were accurate 9344 times. If patients have GERD (Node 22), we predicted that they would survive 90·8% of the time. This rule was applied to 5135 patients, and we were accurate 4661 times.

**CTD-ILD Decision tree analysis**


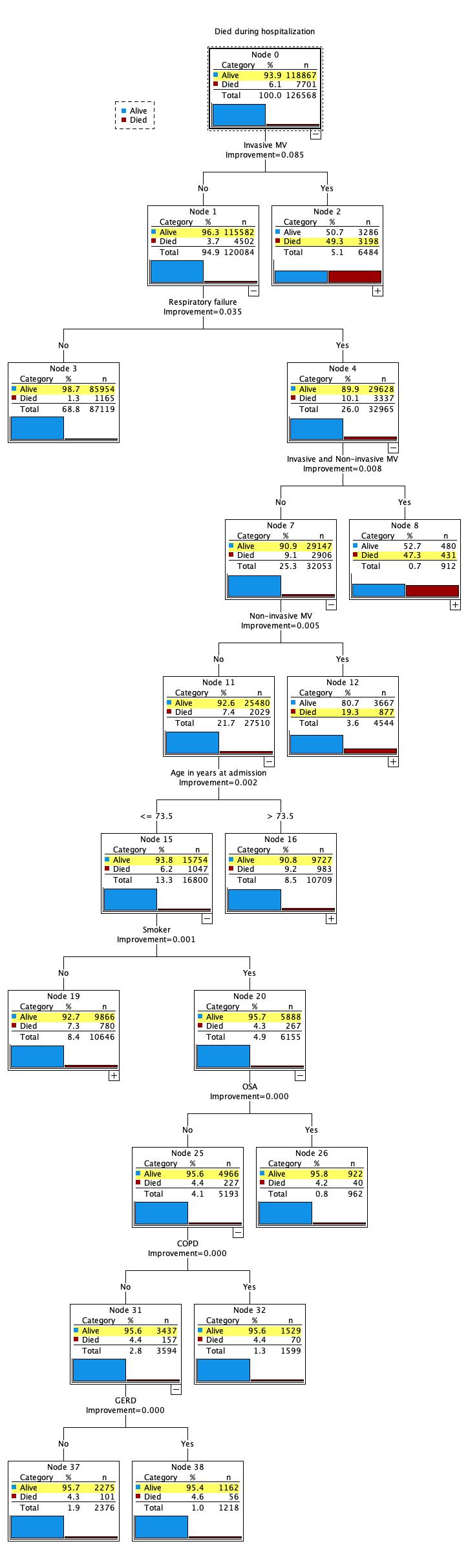


**Figure E7.** Decision tree for CTD-ILD patients’ validation cohort·

In CTD-ILD patients, GERD (nodes 37& 38) was the best predictor in patients with respiratory failure, didn’t receive any mode of MV, didn’t have OSA, COPD, their age are ≤ 73·5 and were not smoker. In this subgroup, if patients didn’t have GERD (Node 37), we predicted that they would survive 95·7% of the time. This rule was applied to 2376 patients, and we were accurate 2275 times. If patients have GERD (Node 38), we predicted that they would survive 95·4% of the time. This rule was applied to 1218 patients, and we were accurate 1162 times.


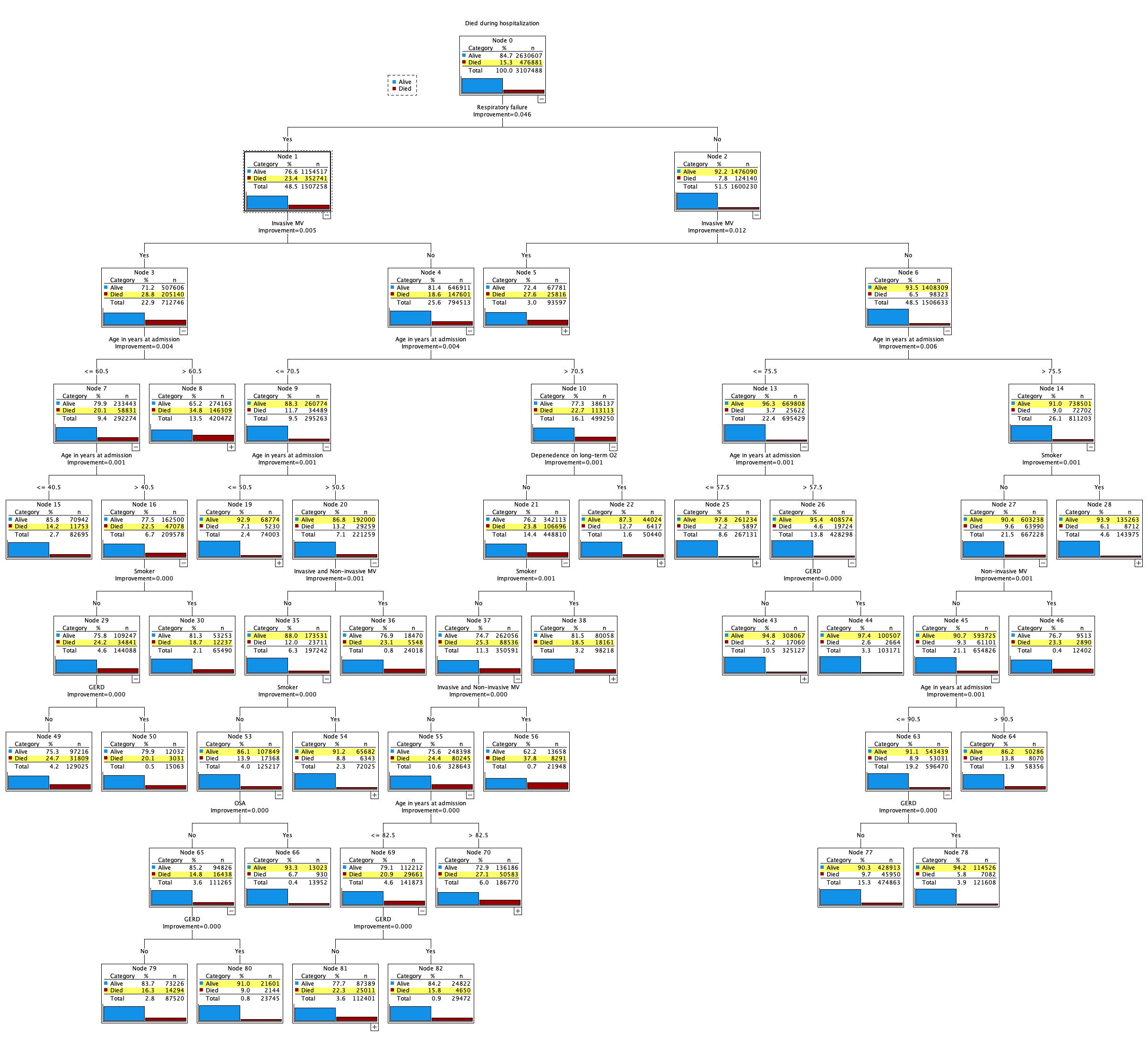
**HP Decision tree analysis**

**Figure E8.** Decision tree for HP patients’ validation cohort.

In HP patients, GERD (nodes 49& 50) was the best predictor in patients with respiratory failure, on invasive mechanical ventilation, non-smoker and their age is between 40·5 - 60·5 years. In this subgroup, if patients didn’t have GERD (Node 49), we predicted that they would die 24·7% of the time. This rule was applied to 129025 patients, and we were accurate 31809 times. If patients have GERD (Node 50), we predicted that they would die 20·1% of the time. This rule was applied to 15063 patients, and we were accurate 3031 times· Besides, GERD (nodes 79& 80) was the best predictor in patients with respiratory failure, didn’t receive any mode of MV, didn’t have OSA, non-smoker, and their age is between 50·5-70·5 years. In this subgroup, if patients didn’t have GERD (Node 79), we predicted that they would die 16·3% of the time. This rule was applied to 87520 patients, and we were accurate 14294 times. If patients have GERD (Node 80), we predicted that they would survive 91% of the time. This rule was applied to 23745 patients, and we were accurate 21601 times. Also, GERD (nodes 81& 82) was the best predictor in patients with respiratory failure, didn’t receive any mode of MV, their age is between 70·5-82·5 years and were not smoker. In this subgroup, if patients didn’t have GERD (Node 81), we predicted that they would die 22·3% of the time. This rule was applied to 112401 patients, and we were accurate 25011 times. If patients have GERD (Node 82), we predicted that they would die 15·8% of the time. This rule was applied to 29472 patients, and we were accurate 4650 times.

In another branch, GERD (nodes 43& 44) was the best predictor in patients without respiratory failure, didn’t receive invasive MV, and their age is between 57·5-75·5 years. In this subgroup, if patients didn’t have GERD (Node 43), we predicted that they would survive 94·8% of the time. This rule was applied to 325127 patients, and we were accurate 308067 times. If patients have GERD (Node 44), we predicted that they would survive 97·4% of the time. This rule was applied to 103171 patients, and we were accurate 100507 times. Finally, GERD (nodes 77& 78) was the best predictor in patients without respiratory failure, didn’t receive any mode of MV, their age is between 75·5-90·5 and were not smoker. In this subgroup, if patients didn’t have GERD (Node 77), we predicted that they would survive 90·3% of the time. This rule was applied to 474863 patients, and we were accurate 428913 times. If patients have GERD (Node 78), we predicted that they would survive 94·2% of the time. This rule was applied to 121608 patients, and we were accurate 114526 times.


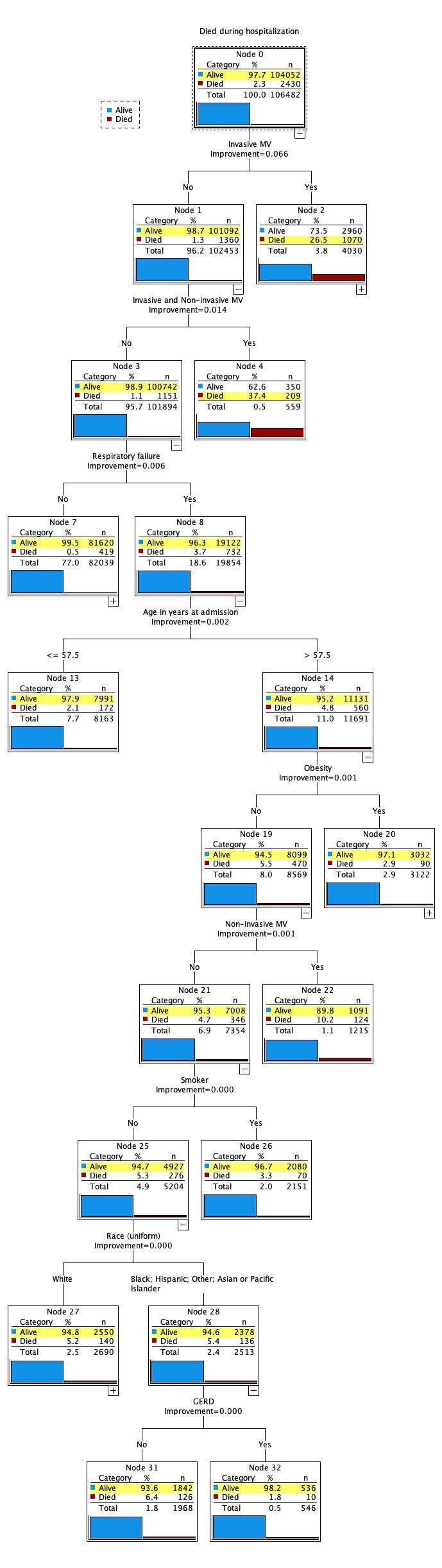
**Pulmonary sarcoidosis Decision tree analysis**

**Figure E9.** Decision tree for pulmonary sarcoidosis patients’ validation cohort.

In pulmonary sarcoidosis patients, GERD (nodes 31& 32) was the best predictor in patients with respiratory failure, didn’t receive any mode of MV, didn’t have OSA, non-obese, non-smoker, non-white and their age are >57·5. In this subgroup, if patients didn’t have GERD (Node 31), we predicted that they would survive 93·6% of the time. This rule was applied to 1968 patients, and we were accurate 1842 times. If patients have GERD (Node 32), we predicted that they would survive 98·2% of the time. This rule was applied to 546 patients, and we were accurate 536 times.

**Unspecified-ILD Decision tree analysis**


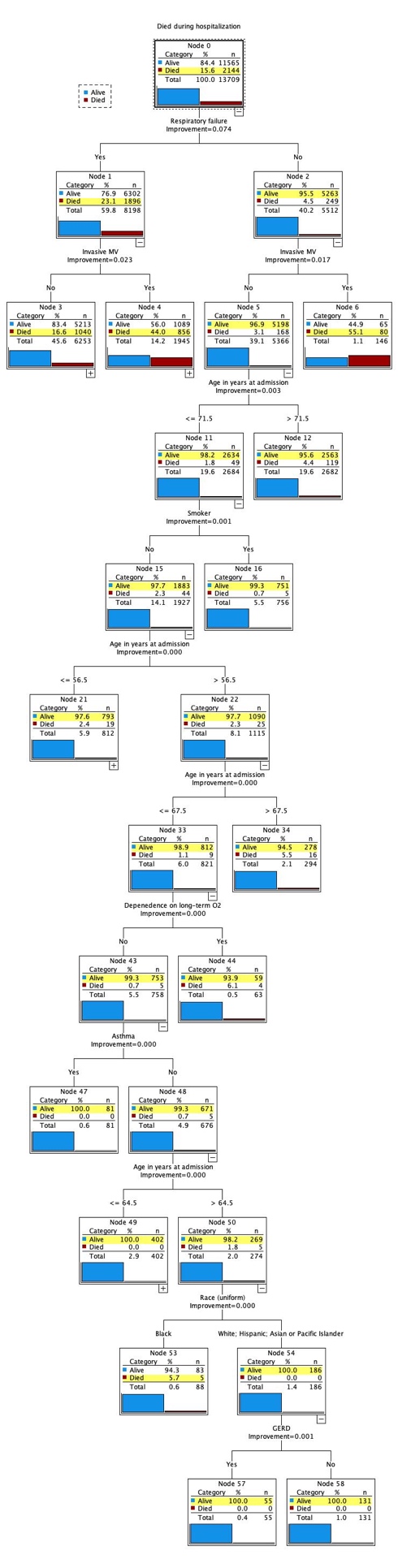


**Figure E10.** Decision tree for Unspecified-ILD patients’ validation cohort.

In patients with unspecified-ILD, GERD (nodes 57& 58) was the best predictor in patients without respiratory failure, didn’t receive invasive MV, didn’t have asthma, not on long term oxygen therapy, non-smoker, non-white and their age is between (67·5-71·5). In this subgroup, if patients didn’t have GERD (Node 58), we predicted that they would survive 100% of the time. This rule was applied to 131 patients, and we were accurate 131 times. If patients have GERD (Node 57), we predicted that they would survive 100% of the time. This rule was applied to 55 patients, and we were accurate 55 times.

1. HCUP Tools and Software. Healthcare Cost and Utilization Project (HCUP). <https://www.hcup-us.ahrq.gov/tools_software.jsp>. Accessed 12 June 2022.

2. Elixhauser Comorbidity Software for ICD-9. Healthcare Cost and Utilization Project (HCUP). <https://www.hcup-us.ahrq.gov/toolssoftware/comorbidity/comorbidity.jsp>. Accessed 12 June 2022.

3. Elixhauser Comorbidity Software for ICD-10. Healthcare Cost and Utilization Project (HCUP). <https://www.hcup-us.ahrq.gov/toolssoftware/comorbidityicd10/comorbidity_icd10.jsp>. Accessed 12 June 2022.
